# Decoupling Activation and Preservation: Architectural Design Principles of Organic Frameworks for Selective Photocatalytic Methane Oxidation

**DOI:** 10.1002/adma.74071

**Published:** 2026-07-15

**Authors:** Minxian Zhang, Jie He, Jinqiang Zhang, Hongqi Sun, Shaobin Wang, Xiaoguang Duan

**Affiliations:** ^1^ School of Chemical Engineering Adelaide University Adelaide South Australia Australia; ^2^ School of Molecular Sciences The University of Western Australia Perth WA Australia

**Keywords:** charge transfer topology, microenvironment engineering, organic frameworks, photocatalytic methane conversion, reactive oxygen species

## Abstract

Photocatalytic methane (CH_4_) conversion faces a dilemma, where C─H bond activation requires a substantial oxidative driving force, whereas partially oxidized C_1_ products are intrinsically more susceptible to further oxidation. As a result, strengthening photocatalytic throughput while avoiding overoxidation remains challenging in the field of highly valuable CH_4_ conversion. Organic semiconductor platforms, including polymeric carbon nitrides (PCN), metal–organic frameworks (MOFs), and covalent organic frameworks (COFs), exhibit structural features that fundamentally differ from those of conventional inorganic counterparts. Their electronic states originate from molecular building units, and their frameworks allow controlled positioning of redox centers, modulation of charge‐transfer pathways, and definition of pore environments, enabling systematic regulation of band energetics, carrier distribution, oxygen activation modes, and proton management of photocatalysts. This review examines how electronic structure engineering, interfacial charge routing, reactive species control, and microenvironment design influence pathway selection in CH_4_ photooxidation. Particular attention is given to mechanisms that favor associative multi‐electron oxygen reduction or energy‐transfer processes while suppressing hydroxyl radical (^•^OH)‐dominated chemistry. Although CH_4_‐specific systems remain limited, mechanistic insights from related photocatalytic reactions provide transferable design principles. Establishing quantitative relationships between architectural parameters and reactive‐species identity is essential for advancing selective solar‐driven C_1_ oxidation.

## Graphical abstract

Selective methane (CH_4_) photooxidation emerges from architectural decoupling of activation and preservation. Programmable organic semiconductor frameworks spatially organize oxidative flux and intermediate residence, separating reaction zones from product domains to synchronize activation strength with stabilization and establish a selective oxidation window.

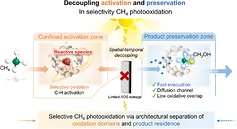

## 1 Introduction

Methane (CH_4_) is the principal component of natural gas and an abundant carbon resource [1, 2]. Conversion of CH_4_ into value‐added C_1_ oxygenates such as methanol (CH_3_OH) and formaldehyde (HCHO) under mild conditions remains a longstanding objective in catalysis [3]. The difficulty of this transformation arises from two intrinsic properties. First, CH_4_ possesses a highly symmetric tetrahedral structure and a C─H bond dissociation energy of approximately 440 kJ mol^−1^, which necessitates strong oxidizing conditions for activation [4, 5]. Second, once partially oxidized products are generated, their weaker C─H bonds and higher polarity render them kinetically more reactive than CH_4_, making secondary oxidation thermodynamically and kinetically favorable [6]. This imbalance between activation and preservation defines the central selectivity challenge in CH_4_ chemistry.

Photocatalysis offers a thermodynamically attractive strategy for CH_4_ conversion as photogenerated charge carriers can drive redox reactions under ambient conditions using solar energy [7, 8]. Studies on metal oxides such as titanium dioxide (TiO_2_) and zinc oxide (ZnO) have demonstrated measurable CH_4_‐to‐oxygenates conversion under light irradiation. Modulation of cocatalysts, crystal facets, and defect states has improved charge separation and altered oxygen activation pathways [9, 10, 11, 12, 13, 14, 15]. Nevertheless, overoxidation frequently persists. Mechanistic investigations indicate that highly reactive oxygen species (ROS), particularly ^•^OH, dominate in many oxide systems. As ^•^OH exhibits near diffusion‐controlled hydrogen abstraction kinetics, they provide minimal discrimination between CH_4_ and partially oxidized intermediates, leading to rapid conversion of CH_3_OH and HCHO to carbon dioxide (CO_2_) even at low overall CH_4_ oxidation [16, 17]. These observations suggest that insufficient control over reactive species identity, rather than inadequate oxidative power, constitutes the primary limitation. In many inorganic photocatalysts, electronic structure, surface coordination environment, and interfacial microenvironment are not independently programmable and thus cannot be optimized in a coordinated manner [18, 19]. Oxygen activation, charge transport, and intermediate diffusion are consequently governed by loosely coupled structural variables [20].

Organic semiconductor materials introduce a different structural paradigm. In polymeric carbon nitride (PCN), metal‐organic frameworks (MOFs), and covalent organic frameworks (COFs), frontier electronic states are defined by covalent organic building units and periodic organization [21, 22]. Meanwhile, donor–acceptor (D–A) motifs, heteroatom substitution, and ionic modulation permit systematic adjustment of band positions and excited‐state characteristics. Besides, reticular architectures allow spatial separation of redox domains and periodic placement of coordination‐defined active sites. Ordered porosity further provides control over adsorption geometry, hydrogen‐bond organization, and proton‐coupled electron transfer (PCET) kinetics. Recent advances in organic photocatalysts‐triggered peroxide synthesis, selective alcohol oxidation, and CO_2_ reduction demonstrate that oxygen activation pathways can be biased toward two‐electron reduction or energy‐transfer mechanisms under appropriate structural conditions [23, 24, 25, 26]. These mechanistic precedents indicate that CH_4_ selectivity may likewise depend on integrated regulation of electronic structure, charge routing, reactive species generation, and microenvironmental constraints.

Despite the growing research on photocatalytic CH_4_ oxidation, most reviews focus on activity enhancement, material optimization, or individual mechanistic case analyses. A systematic perspective that dissects how CH_4_ selectivity is encoded at multiple structural levels, particularly within organic semiconductor architectures, remains underdeveloped. In particular, the interplay between electronic structure, charge topology, reactive species identity, and microenvironmental confinement has not yet been integrated into a unified architectural framework. As such, this review analyzes how these architectural parameters govern pathway selection in CH_4_ photooxidation. After outlining the structural and electronic characteristics of major organic semiconductor platforms, the discussion focuses on four interrelated levels of regulation, including electronic structure, charge architecture, reactive species identity, and microenvironment effects. Emerging CH_4_ photooxidation systems are finally elucidated within this architectural framework, highlighting design implications and open mechanistic questions.

## 2 Fundamentals and Challenges of Photocatalytic CH_4_ Conversion

Photocatalytic CH_4_ conversion follows the manner in which energetic charge carriers are excited and involved in chemically defined reactive intermediates at the catalyst interface. The decisive factor lies in how electrons and holes participate in oxygen activation, hydrogen abstraction, and the stabilization of intermediates. Basically, CH_4_ activation requires oxidative potentials capable of cleaving a C─H bond with a dissociation energy of approximately 440 kJ mol^−1^. In semiconductor‐based photocatalysts, this requirement can be achieved by photogenerated holes or by reactive oxygen species derived from interfacial oxygen reduction. However, under oxidative conditions, CH_3_OH, HCHO, and other partially oxidized intermediates can also undergo rapid oxidation. Consequently, CH_4_ photoconversion efficiency is also determined by reaction control rather than solely by charge‐generation efficiency.

### 2.1 Principles of Photocatalytic CH_4_ Conversion

**FIGURE 1:**
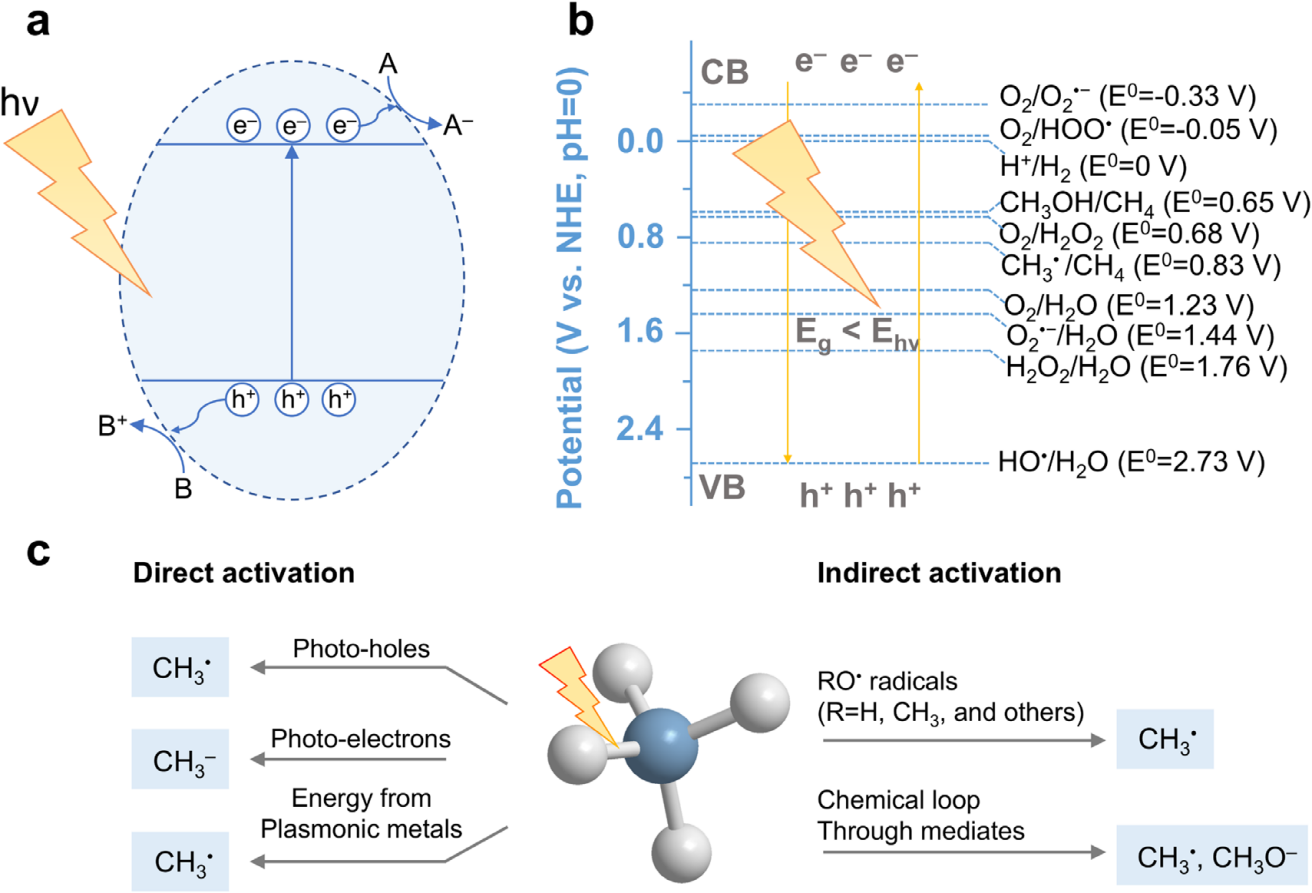
(a) Principle of semiconductor‐based photocatalysis. (b) Potential diagrams of photocatalytic selective CH_4_ oxidation. (c) Different photocatalytic pathways for CH_4_ activation. Reproduced with permission [7]. Copyright 2022, Springer Nature.

Under irradiation, excitation of a semiconductor produces electron‐hole pairs. A fraction of these carriers recombine rapidly, while the remaining carriers migrate to surface sites where redox reactions occur (Figure 1a,b) [27, 28]. The reaction between charges and CH_4_ can take several different pathways (Figure 1c). In the direct hole‐driven activation mechanism, high‐valent surface holes dissociate C─H bond to generate surface‐bound methyl species or methyl species (^•^CH_3_) [29, 30]. This pathway is frequently observed in wide‐bandgap metal oxides [31, 32]. The feasibility of this mechanism depends on the valence‐band position and on the kinetics of hole transfer relative to recombination. In contrast, electron‐mediated pathways are less common but have been proposed under conditions in which photogenerated electrons weaken or cleave C─H bonds via reductive pathways [33]. Plasmonic metals (e.g., Cu, Ag, and Rh) can also promote C─H bond activation via localized surface plasmon resonance. Under light irradiation, localized surface plasmon resonance enhances light harvesting and induces the generation of energetic hot carriers that can be injected into the molecular orbitals of CH_4_ molecules, leading to the weakening and activation of the inert C─H bond [34, 35]. In addition to direct activation, indirect activation mechanisms mediated by reactive intermediates are frequently reported. Among these, radical‐mediated processes dominate most reported systems, where CH_4_ is activated by reactive species such as ^•^OH, ^•^CH_3_, superoxide radical (O_2_^•−^), or hydroperoxyl radical (^•^OOH) generated through charge interactions with water, oxygen, or added oxidants [36, 37, 38, 39, 40].

Alternatively, mediator‐assisted chemical looping mechanisms utilize lattice oxygen or redox‐active metal centers as the primary oxidizing agents, thereby decoupling CH_4_ activation from oxidant regeneration and enabling stepwise oxidation cycles [41, 42]. In this pathway, CH_4_ is activated by localized oxidizing sites associated with lattice oxygen or redox‐active metal centers, forming surface‐bound intermediates while the mediator or catalyst lattice is reduced. The reduced mediator is subsequently regenerated under illumination through reaction with oxygen or other oxidants, completing a cyclic redox process. The mechanism differs from radical‐mediated or direct hole‐driven pathways. Rather than generating freely diffusing reactive species, oxidation proceeds with confined coordination environments, where reactive intermediates remain associated with the catalyst surface. As a consequence, CH_4_ activation and oxidant regeneration can be temporally and spatially separated, thereby suppressing radical propagation in the surrounding medium. Such stepwise processes can improve selectivity by limiting the exposure of partially oxidized products to highly reactive species. By localizing oxidative chemistry at defined active sites and separating CH_4_ activation from product overoxidation, mediator‐assisted pathways can suppress uncontrolled secondary oxidation and favor selective oxygenate formation.

### 2.2 Challenges of Photocatalytic CH_4_ Conversion

The principal constraint in CH_4_ photooxidation arises from competition between primary C─H activation and secondary oxidation of products, as CH_3_OH and HCHO possess weaker C─H bonds and stronger adsorption interactions with polar surfaces than CH_4_. Once formed, these intermediates are frequently re‐exposed to reactive oxygen species prior to desorption [43, 44]. In particular, ^•^OH radical exhibits near diffusion‐controlled hydrogen abstraction kinetics and minimal molecular selectivity. When ^•^OH dominates the oxidation manifold, product preservation becomes intrinsically unfavorable. By contrast, non‐radical oxidants such as singlet oxygen (^1^O_2_), surface‐bound hydroperoxo species, or high‐valent metal–oxo intermediates impose geometric and electronic constraints that can differentiate between CH_4_ and oxygenated products [45]. Selective oxidation depends on the mode of oxygen activation, the kinetics of PCET, and the spatial accumulation of photogenerated charges at catalytic interfaces. In CH_4_ photoconversion, selectivity is further shaped by the effective oxidative strength of the system, the identity of the dominant reactive species, and the residence time of intermediates on the surface. Adjusting a single factor in isolation rarely stabilizes oxygenates, as the oxidative driving force, reactive species formation, and adsorption dynamics evolve simultaneously under illumination. Therefore, improving pathway control requires structural designs that direct charge transport, define oxygen adsorption geometry, and regulate proton transport at the interface.

## 3 Organic Semiconductors as Programmable Photocatalysts

Selective CH_4_ photooxidation requires coordinated regulation of oxidation potential, reactive species identity, carrier distribution, and intermediate exposure. In conventional inorganic oxides, these variables are tightly coupled as band energetics, surface states, and adsorption properties originate from a common extended lattice [46, 47]. Adjustment of one structural parameter frequently perturbs others, constraining independent control over oxygen activation and product preservation [48].

Organic semiconductor architecture provides a distinct structural paradigm. In these systems, electronic structure and catalytic function arise from modular π‐conjugated building units rather than rigid ionic frameworks [49, 50, 51]. Light absorption, band formation, and charge transport are governed predominantly by delocalized organic backbones. These architectures include PCN, COFs, and MOFs, in which linker‐derived states directly contribute to the frontier bands [52, 53]. Although MOFs contain inorganic nodes, many semiconductive variants exhibit band formation and carrier transport dominated by extended organic linkers, supporting their inclusion within this category. The defining advantage of these systems is programmability. Electronic polarization, coordination geometry, and pore environment can be adjusted through molecular design and reticular assembly without disrupting one another in proportion [54, 55]. This structural flexibility enables partial decoupling of variables that are intrinsically linked in dense oxides.

Despite this structural programmability, organic semiconductor photocatalysts still face several barriers that can limit their apparent CH_4_ conversion activity relative to many conventional inorganic photocatalysts. Charge generation, separation, and transport are often constrained by localized excited states, exciton binding, energetic disorder, and interframework hopping, which restrict the migration of oxidative equivalents to CH_4_ activation sites [56, 57]. Moderate light absorption, low steady‐state carrier density, and oxidative instability under reactive oxygen environments may further limit apparent activity and long‐term durability [58]. These material limitations are coupled with the reaction‐specific difficulty of CH_4_ photooxidation. Strong C─H bond requires sufficient oxidative driving force, whereas partially oxidized products such as CH_3_OH and HCHO are more susceptible to further oxidation [59, 60]. Therefore, the key barrier is not only insufficient activity, but the difficulty of synchronizing CH_4_ activation with oxygenate preservation. This challenge provides the rationale for architectural regulation through electronic structure engineering, carrier routing, reactive species control, and microenvironment design.

### 3.1 Electronic Modularity

In π‐conjugated frameworks, band‐edge positions and optical transitions are determined by molecular orbital interactions [61]. D–A motifs, heteroatom incorporation, and backbone planarity allow systematic tuning of redox potentials while preserving the overall scaffold [62, 63]. π–π interactions further influence charge migration and separation, shaping photogenerated carrier dynamics [64]. Such electronic modularity enables adjustment of the oxidation window without reconstructing catalytic cavities. Narrowing the valence‐band potential reduces the driving force for CH_3_OH overoxidation while retaining sufficient oxidative power for CH_4_ activation. In semiconductive MOFs, functionalization of organic linkers modulates band structure independently of node connectivity, offering additional control over redox energetics [65]. This separation between band engineering and site geometry distinguishes organic semiconductor architectures from many inorganic oxides, where band tuning often alters surface chemistry simultaneously [48]. Therefore, electronic modularity defines the energetic latitude of CH_4_ oxidation by positioning the valence band within a constrained redox window, enabling activation without proportionally amplifying overoxidation risk.

### 3.2 Reticular Precision

A second structural advantage of organic semiconductor architectures is reticular precision, which provides defined spatial separation and coordination environments for active sites [51, 66]. In COFs, covalent linkage of organic monomers into periodic frameworks creates well‐ordered pores and crystallinity that facilitate non‐random adsorption and diffusion of reactants [67]. In PCN, extended triazine‐based networks and nitrogen‐rich cavities serve as anchor points for isolated metal atoms or multinuclear motifs [68, 69]. This precise placing of active centers supports geometric confinement of oxygen activation sites, which is a prerequisite for pathway‐defined selectivity. Semiconductive MOFs inherently separate metal nodes through organic linkers, affording site isolation that limits metal aggregation and enables cooperative multi‐site configurations [70, 71, 72]. Defined coordination environments influence how molecular oxygen binds and transforms. Geometrically constrained pockets can stabilize associative oxygen intermediates and restrict diffusive radical propagation. Reticular precision, therefore, reshapes the reactive manifold at the level of oxygen activation rather than relying solely on radical suppression.

### 3.3 Microenvironment Capability

**FIGURE 2:**
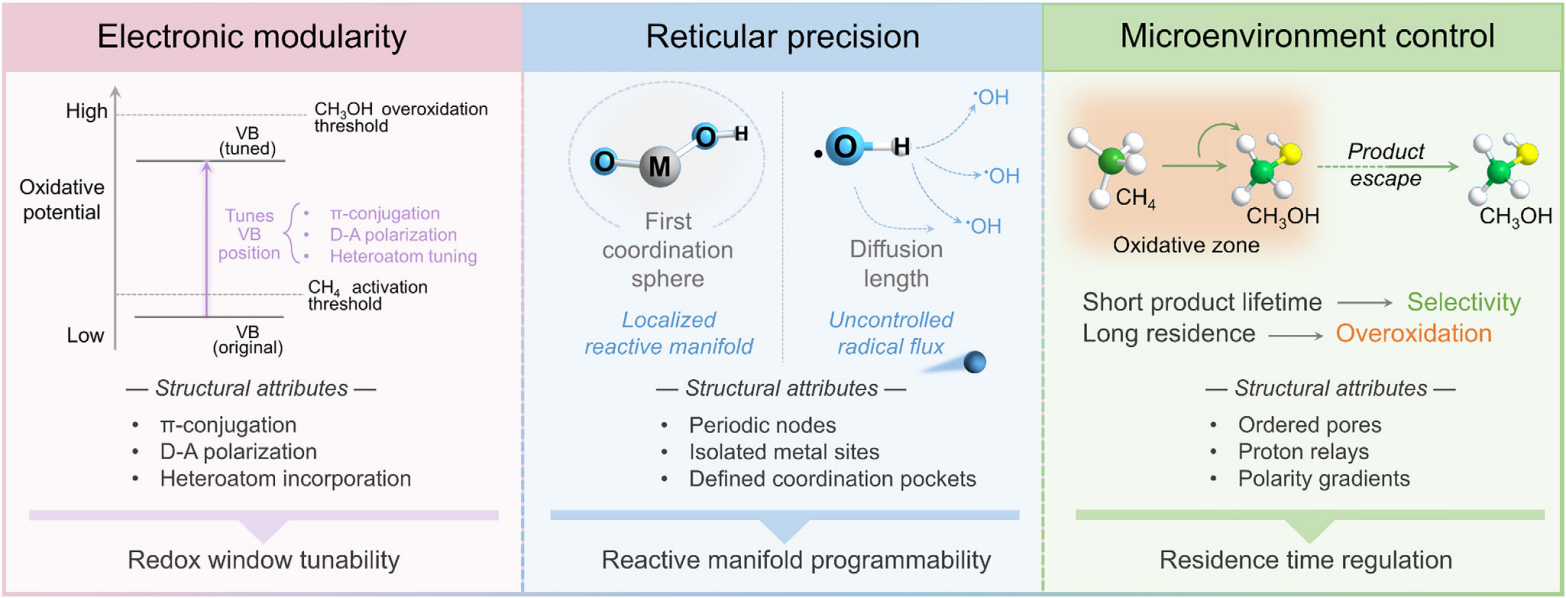
Structural programmability of organic semiconductor photocatalysts.

Beyond electronic and coordination variables, organic semiconductor frameworks offer tunable microenvironments defined by pore polarity, linker functionality, and hierarchical porosity. These structural features regulate adsorption strength, diffusion pathways, and the stabilization of intermediates [73, 74]. Polar functional groups and hydrogen‐bond networks can influence local proton‐transfer kinetics and modulate interactions between partially oxidized intermediates and oxidative domains. Hierarchical pore structures in COFs and PCNs shorten diffusion paths and facilitate oxygenate removal [75, 76]. Likewise, membrane‐integrated MOF architectures demonstrate how gas‐solid interfaces can enhance CH_4_ accessibility while limiting product reoxidation [77]. Programmable microenvironments provide a structural basis for residence‐time control. CH_3_OH can be generated in one region and transported away from oxidative equivalents before undergoing secondary transformation. Spatial organization of adsorption and desorption domains further complements electronic and coordination design by controlling intermediate residence and product evacuation. Electronic structure, coordination geometry, and microenvironmental organization in organic semiconductor frameworks arise from modular molecular units rather than from a unified inorganic lattice [78, 79]. This modularity enables a higher degree of separation between band energetics and interfacial chemistry than is typically accessible in conventional transition‐metal oxides. Microenvironmental design further regulates the temporal fate of partially oxidized intermediates by balancing their residence against local oxidative flux, thereby supporting activation–preservation decoupling. These structural attributes define the architectural programmability of organic semiconductor photocatalysts, as summarized in Figure 2.

### 3.4 Mechanistic Validation Under CH_4_ Conversion Conditions

The programmability of organic semiconductor photocatalysts must be evaluated not only through structural and electronic characterization, but also through reaction‐specific mechanistic validation under CH_4_ conversion conditions. This requirement is particularly significant as CH_4_ activation, reactive species generation, product preservation, and catalyst stability occur simultaneously under light irradiation. In situ and operando spectroscopic techniques provide direct evidence for the reactive pathways involved in CH_4_ photooxidation. In situ Fourier transform infrared spectroscopy (FTIR) or diffuse reflectance infrared Fourier transform spectroscopy (DRIFTS) can track surface‐bound intermediates, including methyl, methoxy, hydroperoxo, and carbonyl‐containing species, thereby clarifying whether CH_4_ oxidation proceeds through sequential oxygenation, radical‐mediated pathways, or coordination‐confined intermediates. Electron paramagnetic resonance (EPR) spectroscopy with spin‐trapping agents enables the identification of reactive species, distinguishing radical pathways involving ^•^OH or O_2_^•−^ from non‐radical routes such as ^1^O_2_, metal‐oxo, or surface‐bound oxygen intermediates. Transient absorption spectroscopy and time‐resolved photoluminescence provide additional insights into excited‐state dynamics, charge separation, and carrier lifetimes, thereby linking molecular design to charge utilization during oxygen activation.

For organic photocatalysts, product‐origin verification is equally significant, as these materials contain carbon‐rich frameworks; the apparent formation of C_1_ products may be complicated by framework degradation or carbon leaching under oxidative photocatalytic conditions. Isotopic labeling experiments using ^13^CH_4_, coupled with ^13^C nuclear magnetic resonance (NMR), or gas chromatography‐mass spectrometer (GC‐MS), are therefore critical to confirm that carbon‐containing products such as CH_3_OH, HCHO, formic acid (HCOOH), or CO_2_ originate from CH_4_ rather than from the organic frameworks. Complementary post‐reaction characterization, including FTIR, X‐ray photoelectron spectroscopy (XPS), solid‐state NMR, elemental analysis, and structural analysis, should be used to assess whether the framework and active sites remain intact after photocatalytic reaction. Adsorption and desorption measurements, including CH_4_‐temperature programmed desorption (CH_4_‐TPD) and CH_3_OH‐temperature programmed desorption (CH_3_OH‐TPD), can further clarify substrate accessibility and product residence behavior. These measurements are particularly relevant to organic frameworks with tunable pores and hydrophobicity, where CH_4_ enrichment and oxygenate desorption strongly influence selectivity. Together, these characterization strategies establish the experimental basis for correlating organic photocatalyst structure with CH_4_ activation pathways, reactive species identity, product origin, and catalytic stability.

## 4 Architectural Regulation of CH_4_ Photooxidation

**FIGURE 3:**
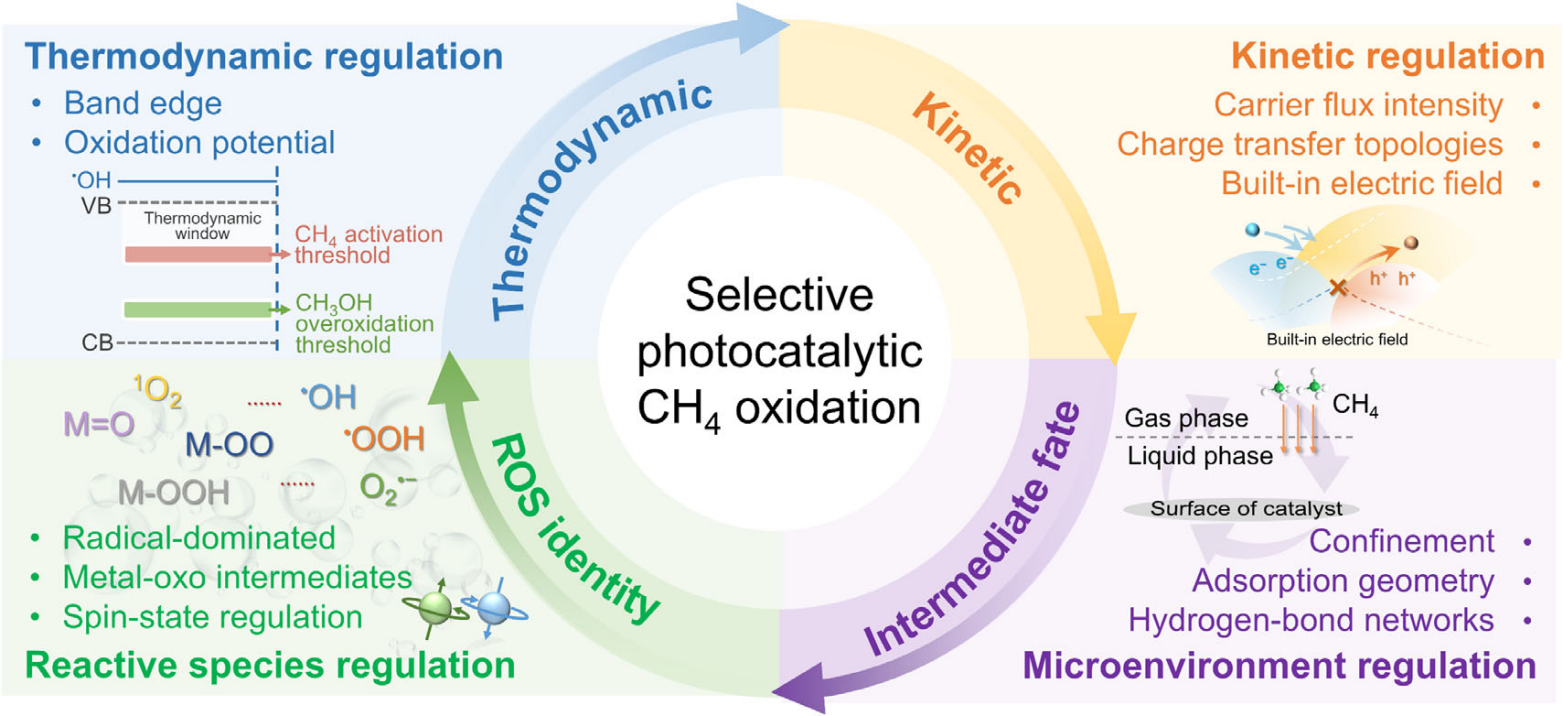
Architectural framework governing selective CH_4_ photooxidation over organic semiconductor photocatalysts.

Selective CH_4_ photooxidation is governed by the architectural organization of four interdependent dimensions, including oxidation potential, carrier routing, reactive‐species identity, and intermediate residence. These variables define (i) the thermodynamic window for C─H activation, (ii) the spatial distribution of redox equivalents, (iii) the mode of oxygen activation, and (iv) the lifetime of partially oxidized intermediates. As illustrated in Figure 3, these factors are not independent control parameters but mutually constrained outcomes of framework architecture. In organic semiconductor systems, the arrangement of π‐conjugated backbones, coordination geometries, and pore domains dictates how energetic driving force, reactive flux, and mass transport are integrated. Therefore, selectivity emerges not from a single optimized variable but from architectural regulation that synchronizes activation with preservation.

### 4.1 Thermodynamics Regulation

**FIGURE 4:**
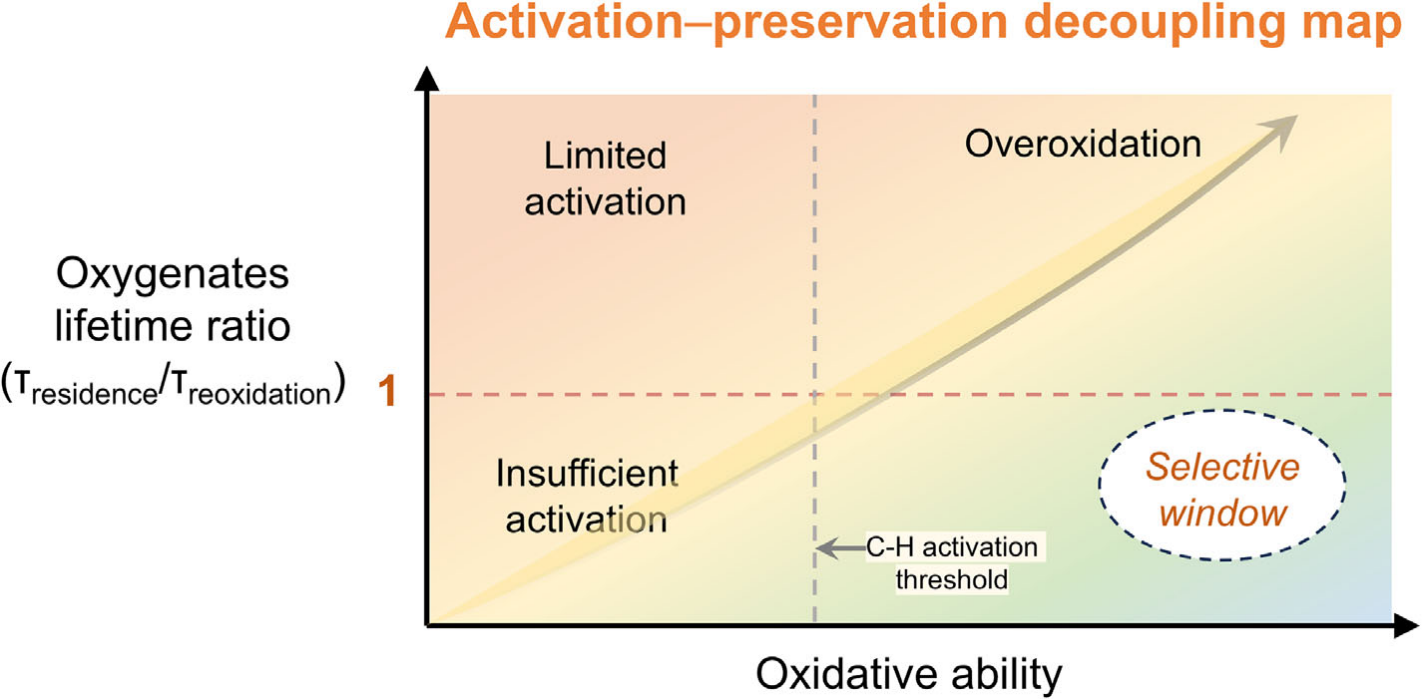
Thermodynamic activation–preservation decoupling map for selective CH_4_ photooxidation. The product lifetime ratio (T_residence_/T_reoxidation_) defines the kinetic competition between intermediate escape and reoxidation. Values greater than 1 indicate that reoxidation outpaces product escape, favoring overoxidation; a value of 1 corresponds to kinetic parity; values below 1 signify that intermediates exit the oxidative domain more rapidly than they are reoxidized, enabling product preservation when the activation threshold is met.

Thermodynamic regulations set the energetic boundary for selective CH_4_ photooxidation. Selective conversion requires oxidative strength sufficient for C─H activation while maintaining the activation–preservation decoupling condition illustrated in Figure 4. This requirement confines CH_4_ photooxidation to a narrow thermodynamic window, in which the oxidation potential enables C─H bond cleavage without accelerating CH_3_OH reoxidation. This selective regime reflects an intrinsic energetic asymmetry between CH_4_ activation and oxygenate preservation. Electronic structure determines whether photogenerated holes are positioned within this window.

In organic semiconductor frameworks, frontier orbital energies arise from π‐conjugated backbones and molecular polarization motifs, enabling band positions to be tuned without reconstructing the catalytic scaffold [80, 81, 82]. Thermodynamic regulation extends beyond bandgap modification, requiring oxidative strength to be positioned within a defined energetic interval and spatially distributed within the lattice. Structural strategies such as π‐conjugation extension, D–A polarization, heteroatom incorporation, and ionic‐state modulation shift or redistribute frontier orbitals [57, 83, 84]. Their significance for CH_4_ conversion depends on whether hole potentials are aligned with the activation threshold while maintaining tolerance toward partially oxidized intermediates.

#### 4.1.1 Oxidation Threshold

C─H bond activation in CH_4_ requires an oxidation potential capable of overcoming a bond dissociation energy of approximately 440 kJ mol^−1^, whereas CH_3_OH oxidation proceeds at lower energetic thresholds. This energetic mismatch creates an intrinsic asymmetry in selective CH_4_ photoconversion. Increasing hole potential facilitates CH_4_ activation but simultaneously narrows the stability margin for oxygenated products. Selective CH_4_ oxidation therefore requires not simply stronger oxidation, but precise alignment of the valence‐band position within a narrow energetic interval that enables C─H activation without accelerating secondary oxidation.

Modification of the π‐conjugated skeleton provides a strategy for such alignment, as it alters the valence‐band character without disrupting the overall framework. Nitrogen‐enriched triazole‐based carbon nitride (C_3_N_5_) exhibits redistributed π‐electron density and internal polarization that modify hole localization and redox energetics [85]. This electronic reorganization illustrates how conjugation engineering can bias oxidation strength towards defined lattice regions rather than increasing it uniformly. For CH_4_ conversion, such spatial differentiation may confine high‐energy holes to specific lattice sites and reduce secondary oxidation of partially oxidized products. An analogous principle applies to reticular frameworks, where conjugation length can be independently tuned from node topology. Bryant et al. synthesized a series of Ti_6_O_9_‐based MOFs incorporating oligo‐p‐arylene linkers of increasing conjugation length [86]. Increasing π‐extension narrows the HOMO–LUMO gap and promotes electron transfer from the linker to Ti node while preserving geometric site isolation. As orbital redistribution modifies the oxidation potential without altering the coordination architecture, thermodynamic alignment can be achieved without compromising the structural definition of active sites. Furthermore, COFs demonstrate framework‐level control over orbital alignment. Chen and co‐workers designed COFs in which π‐density and pore structure were optimized to spatially separate HOMO and LUMO distributions [87]. This electronic partitioning localized oxidation and reduction sites within a crystalline lattice and enabled coupled redox reactions without global bandgap contraction. When oxidative density is confined to defined regions, overlap between strongly oxidizing holes and oxygenated intermediates is reduced, which is critical for suppressing overoxidation during CH_4_ conversion. Chemical‐state modulation in CN highlights the sensitivity of this alignment. Stölting and co‐workers showed that hydrofluoric acid treatment of CN induces reversible adaptive states, accompanied by measurable changes in band structure [88]. Exfoliation‐induced thinning has likewise been correlated with systematic shifts in optical absorption and electronic properties, as reported by Huang and co‐workers [89]. These observations illustrate that moderate displacement of the valence‐band position can determine whether hole potentials remain within the activation threshold for CH_4_ or become sufficiently positive to promote overoxidation. These examples highlight that oxidation‐threshold engineering is useful only when it provides energetic precision rather than indiscriminately increasing oxidative strength.

#### 4.1.2 Oxidative Density Distribution

Thermodynamic regulation in CH_4_ photooxidation depends not only on the absolute position of the valence band, but also on the spatial distribution of oxidative strength within the catalytic lattice. Uniform deepening of the valence band increases oxidative bias across the surface and reduces tolerance toward partially oxidized intermediates [90]. In contrast, spatially confined hole density restricts high‐energy oxidation to defined lattice regions, lowering the probability that CH_3_OH encounters equivalent oxidative potential immediately after formation [91]. Selectivity benefits from redistributing oxidative strength rather than enhancing it indiscriminately.

D–A polarization provides an effective strategy for spatial control by separating electron and hole densities in the excited state. Chen and co‐workers introduced a propeller‐like donor unit (TAPT) into polymeric CN through copolymerization with triazine acceptor units, generating asymmetric charge‐density distributions as confirmed by density functional theory (DFT) calculations and transient absorption spectroscopy [92]. The resulting electronic polarization localized hole density preferentially at defined molecular motifs instead of distributing it uniformly throughout the lattice. In CH_4_ systems, this spatial differentiation reduces overlap between high‐energy holes and oxygenated intermediates that adsorb in adjacent regions. Periodic D–A ordering in crystalline frameworks further refines this redistribution. Qin et al. reported dual D–A COFs featuring spatially separated redox centers that enabled directional charge migration and selective two‐electron oxygen reduction [83]. In situ EPR measurements linked ordered D–A polarity with stabilized charge‐transfer states and suppressed radical formation. D–A coupling also influences excited‐state relaxation pathways, which determine how long oxidative strength persists. Wang et al. constructed host–guest D–A MOFs that promoted intersystem crossing and favored energy transfer to O_2_ by modulating relaxation dynamics [93]. Redistribution of excited‐state character governs both the spatial location and temporal persistence of oxidative equivalents. D–A polarization benefits CH_4_ selectivity only when redistributed hole potentials remain within the activation–preservation window. Excessive stabilization of charge‐transfer states may lower hole energy below the threshold required for C─H activation, whereas overly strong donor incorporation can deepen the valence band and compress the oxygenate‐preservation margin.

#### 4.1.3 Redox Window Modulation

Heteroatom incorporation alters local frontier‐orbital symmetry and electrostatic potential without reconstructing the overall framework topology [62, 94]. As CH_4_ selectivity is governed by the relative position of the activation and preservation thresholds, even modest changes in valence‐band character can determine whether the energetic window remains compatible with C─H activation while preserving CH_3_OH stability. Unlike global bandgap modulation, which uniformly changes oxidation strength across the surface, heteroatom tuning operates at the orbital level and introduces localized asymmetry in charge density [95]. Sebastian‐Garate and co‐workers demonstrated that the incorporation of B, P, O, and S into CN induces dopant‐dependent redistribution of frontier orbitals and modifies electron‐localization patterns [96]. DFT analysis revealed that specific dopant configurations selectively stabilized two‐electron oxygen reduction pathways. Rather than increasing oxidative bias uniformly, orbital asymmetry confines oxidation strength to selected lattice regions. In CH_4_ systems, this confinement is beneficial only when high‐energy oxidation is restricted to activation‐relevant sites and does not extend to domains where CH_3_OH resides. Single‐atom incorporation represents a more localized form of electronic perturbation. Atomically dispersed Sb species embedded within CN reorganized charge density in the Sb‐N coordination environment and reduced activation barriers for oxygen activation [97]. Operando spectroscopic measurements indicated electron accumulation at discrete coordination motifs without substantial global band shifts. For CH_4_ conversion, this site‐specific modulation can promote formation of high‐valent intermediates at controlled locations while avoiding widespread valence band deepening. Poorly defined coordination environments, however, may introduce additional radical pathways and broaden the effective oxidation range.

In the crystalline framework, heteroatom effects can emerge as periodic polarization. Su et al. incorporated nitro substituents into a metal‐free COF, generating intraframework dipoles that enhanced exciton dissociation and promoted two‐electron oxygen reduction [98]. Kelvin probe measurements confirmed the formation of an internal field, and DFT analysis revealed modified O_2_ adsorption energetics. Periodically organized electronegativity differences can bias oxidative equivalents toward specific lattice regions, reducing direct overlap between newly formed oxygenates and strongly oxidizing holes. Strongly electron‐withdrawing substituents, however, may lower conduction‐band energies and perturb the reductive half‐reaction required for controlled oxygen activation. Thermodynamic alignment must therefore be evaluated on both oxidative and reductive sides of the catalytic cycle.

Heteroatom incorporation in MOFs provides another route to orbital‐level redox tuning. Partial replacement of redox‐inactive Zn(II) with Co(II) in imidazolate frameworks modified ligand‐to‐metal charge‐transfer energetics and increased electron accumulation at metal nodes, while preserving crystallographic order [99]. Adjustment of node‐centered orbitals altered redox alignment without disrupting site isolation. For CH_4_ oxidation, such tuning can influence the formation energy and stability of high‐valent metal–oxo intermediates. Incorporation of redox‐active heterometals may also expand the oxidation range if not precisely controlled, thereby compressing the activation–preservation interval.

Therefore, heteroatom incorporation is better regarded as a strategy for reshaping the energetic window through localized orbital perturbation than as a generic performance enhancer. Its impact on CH_4_ photooxidation depends on whether dopant‐induced electronic asymmetry narrows the interval toward selective C─H activation or broadens it toward uncontrolled overoxidation. Although improved activity is frequently reported after heteroatom modification, quantitative assessment of how dopant‐driven band shifts influence CH_4_ selectivity remains scarce. Direct correlations among local orbital perturbation, hole‐potential distribution, and oxygenate survival probability are essential for rational thermodynamic design.

#### 4.1.4 Ionic‐State Effects

Ionic‐state modulation reshapes local electrostatic environments and effective band alignment without altering the covalent backbone of the framework [100]. Proton activity and local dielectric properties influence the effective oxidation potential experienced at catalytic sites [101]. In CH_4_ systems, such modulation affects both C─H activation and subsequent oxygen‐reduction pathways.

Ion exchange within poly(heptazine imide) lattices modifies redox energetics and charge‐storage behavior. Hattori and co‐workers showed that the replacement of alkali cations in PHI frameworks shifts band‐edge positions and alters photoelectrochemical performance [102]. Counterion redistribution changes electron accumulation and redox alignment, thereby altering the energetic requirements for hole‐driven C─H activation. Excessive electron‐density stabilization decreases the effective oxidation strength and makes CH_4_ activation less favorable, whereas insufficient stabilization may permit deeper valence‐band positions and compress the activation–preservation interval. More dynamic regulation arises from proton‐responsive frameworks. Liu's group reported a squaric acid‐based zwitterionic COF (STT COF) that undergoes spontaneous hydrogenation under irradiation, as supported by in situ FTIR spectroscopy and negligible kinetic isotope differences between H_2_O and D_2_O [103]. In CH_4_ systems, such coupling influences whether oxygen activation proceeds through associative pathways or shifts toward radical‐generating homolysis. However, excessive protonation may deepen valence‐band potentials or alter conduction‐band alignment, narrowing the selective window and accelerating oxygenate oxidation.

In MOFs, ligand protonation and ionic functionalization further modify node‐centered redox behavior and local electric fields. Diamine‐functionalized Zr‐based MOFs have been shown to adjust the energetics of oxygen activation through ligand‐induced electronic redistribution [104]. Protonated ligands altered metal–oxo formation thresholds and affected electron‐transfer kinetics. Band alignment in MOFs arises from the combined electronic contributions of metal nodes and organic linkers; ionic perturbations at either component can shift the absolute redox energetics. Tang et al. demonstrated that replacing Cu with Ag in cyclic trinuclear‐unit MOFs alters band‐edge positions and photocatalytic behavior without major changes in surface area [105]. Such node substitution tunes the formation energy of high‐valent intermediates that may participate in CH_4_ activation. Kolobov and co‐workers emphasized the importance of evaluating MOF photocatalysis using absolute band‐edge descriptors rather than relying solely on molecular analogies [53].

Precise knowledge of absolute band positions is essential because both activation and preservation thresholds must be satisfied simultaneously. Reticular COFs design offers systematic control over band positions at the molecular level. Gu and co‐workers constructed an isoreticular COF series to decouple conjugation and inductive effects, observing predictable variation in band‐edge positions correlated with photocatalytic feasibility [106]. Wang et al. combined first‐principles calculations with machine learning to predict COF band edges directly from precursors [107]. Such predictive strategies are particularly relevant to CH_4_ conversion, where oxidative and reductive limits must be positioned within a constrained energetic interval. Overall, ionic‐state modulation expands thermodynamic control from static band alignment to operation‐dependent electrostatic tuning, but its selectivity benefit depends on whether the activation and preservation thresholds remain simultaneously satisfied.

In summary, thermodynamic regulation defines the energetic boundary of selective CH_4_ photooxidation. Effective architectures must provide sufficient oxidative driving force for C─H activation without globally increasing the probability of oxygenate overoxidation. Central requirement is not to maximize oxidation potential, but to position and localize oxidative strength within an activation‐compatible and preservation‐tolerant window. Once this energetic window is established, carrier kinetics determine how oxidative equivalents are delivered in time and space.

### 4.2 Kinetic Regulation

**FIGURE 5:**
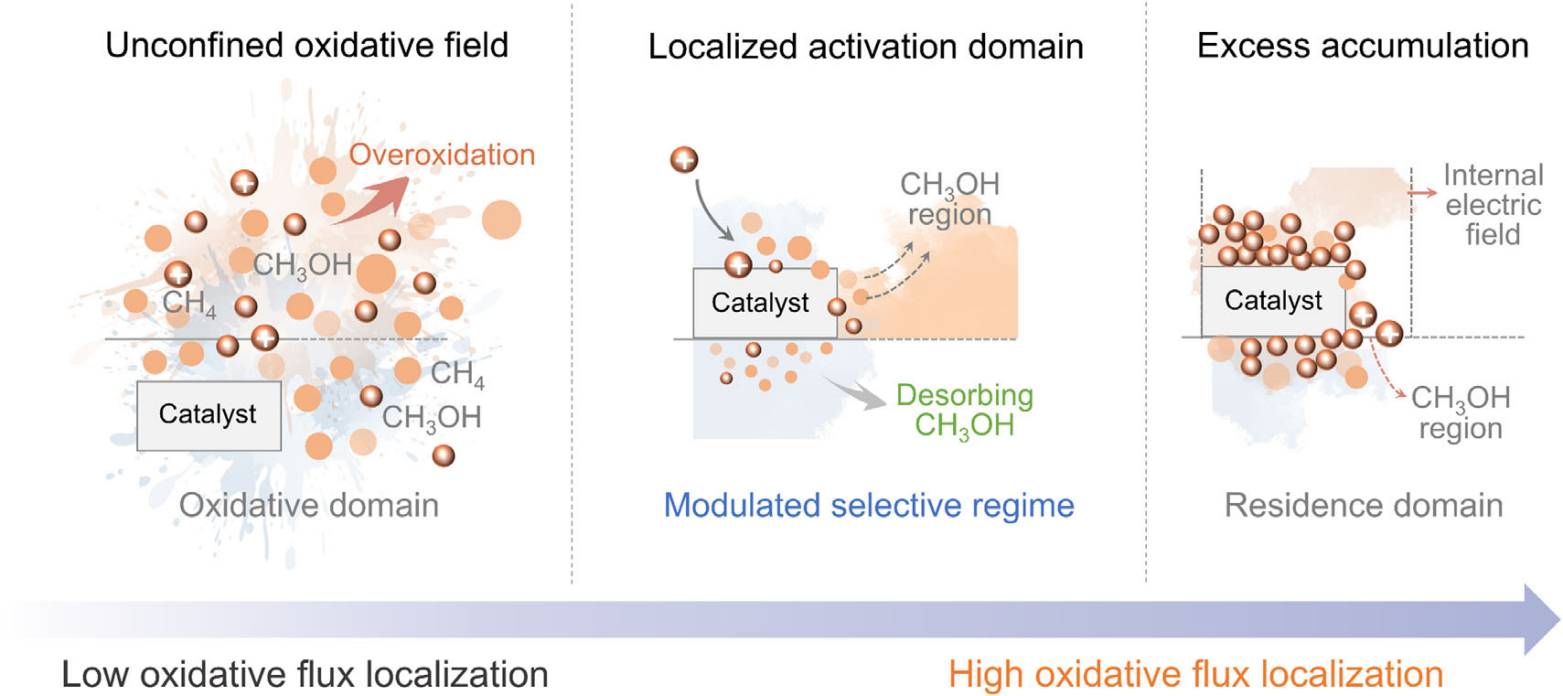
Spatial regulation of oxidative flux in kinetic control of selective photocatalytic CH_4_ oxidation.

Kinetic regulation determines how rapidly and where photogenerated carriers are transferred once CH_4_ activation becomes thermodynamically accessible. Whereas electronic structure defines the energetic feasibility of CH_4_ activation, carrier transfer governs the kinetic efficiency of CH_4_ oxidation and thus influences product yield and selectivity [108]. In CH_4_ photooxidation, C─H activation, oxygen reduction, and CH_3_OH oxidation proceed on distinct kinetic time scales. When the flux of photogenerated holes exceeds the intrinsic stabilization or desorption rate of partially oxidized intermediates, the oxidative field extends beyond the activation domains and the product lifetime shortens [109]. Kinetic regulation requires synchronization of carrier transfer with intermediate stabilization and diffusion. Product selectivity is maintained only when oxidative equivalents accumulate at a rate that does not outpace preservation processes. Interfacial coupling strength, charge‐transfer topology, and internal electrostatic gradients determine how rapidly and where high‐energy carriers accumulate. Their impact on CH_4_ selectivity depends not only on charge‐separation efficiency, but also on whether oxidative flux remains confined within activation‐relevant regions rather than overlapping with CH_3_OH residence domains, as schematically illustrated in Figure 5. The spatial distribution and degree of localization of photogenerated holes determine whether oxidative equivalents intersect with intermediate residence domains. In unconfined oxidative fields, dispersed hole flux overlaps with CH_3_OH accumulation regions and promotes secondary oxidation. Localized activation domains can spatially separate C─H activation from product residence, enabling desorption before overoxidation. However, excessive hole accumulation may re‐intensify local oxidative stress even under geometric confinement. Selectivity is achieved when oxidative flux is spatially confined yet quantitatively moderated to align with the kinetics of intermediate stabilization and diffusion.

#### 4.2.1 Carrier Flux Intensity

Interfacial coupling governs the rate at which photogenerated carriers reach reactive sites and defines the intensity of oxidative flux [110]. Weak coupling limits CH_4_ activation even when thermodynamic alignment is favorable, while excessive coupling can accelerate hole transfer, intensify radical formation, and shorten oxygenate lifetime. The decisive variable is balanced carrier flux compatible with intermediate stabilization, rather than maximal carrier transfer [111, 112].

In van der Waals‐stacked CN/COF heterostructures, π–π interactions enhance electronic communication without requiring lattice matching. Wang and co‐workers constructed a nitrogen‐vacancy‐containing g‐C_3_N_4_ coupled with Tp–Tta COF (g‐C_3_N_4_/Tp–Tta COF) through a solvent‐assisted self‐assembly [113]. Spectroscopic characterization revealed reduced interfacial resistance and faster carrier migration compared with the individual components. Enhanced carrier transfer increases oxidative flux at reactive interfaces and can promote C─H activation, but secondary oxidation becomes increasingly probable when hole accumulation exceeds the rate of CH_3_OH desorption or stabilization. Composite systems further illustrate that coupling strength depends on electronic connectivity rather than contact area. Hassan and co‐workers reported benzothiadiazole–pyrene COF/g‐C_3_N_4_ composites with optimal activity at a composition corresponding to a percolation threshold [114]. Continuous transport pathways across the interface sustained carrier flow and prevented localized accumulation. In CH_4_ conversion, incomplete coupling may generate discrete regions of high oxidative density that accelerate overoxidation, while electronically continuous networks distribute carriers more uniformly and moderate local oxidative bias.

**FIGURE 6:**
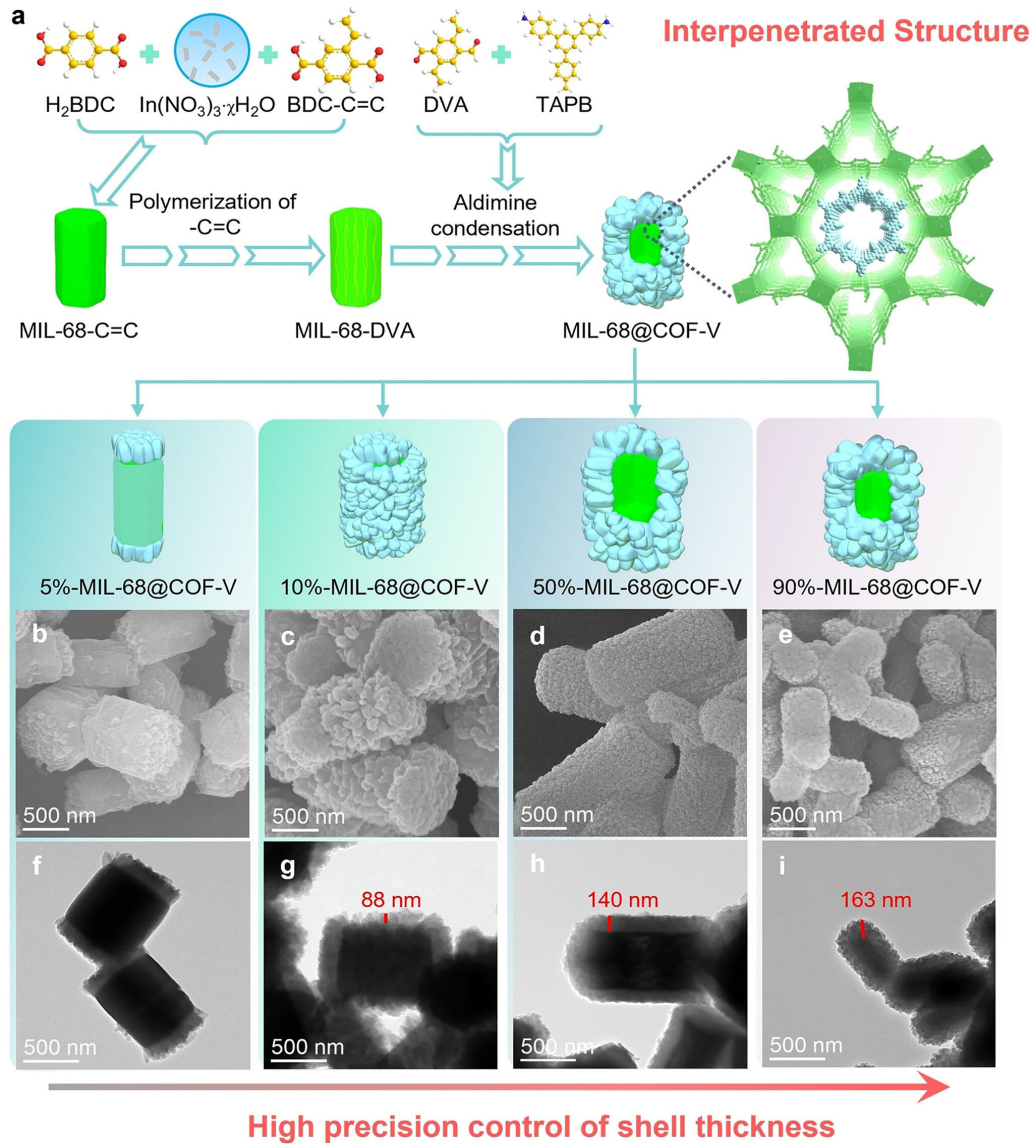
(a) Schematic diagram for the construction of MIL‐68@COF−Vs. (b–i) SEM and TEM images of MIL‐68@COF−Vs, including (b,f) 5 %‐MIL‐68@COF−V, (c,g) 10 %‐MIL‐68@COF−V, (d,h) 50 %‐MIL‐68@COF−V, and (e,i) 90 %‐MIL‐68@COF−V. Reproduced with permission [116]. Copyright 2024, Wiley‐VCH.

Covalently linked interfaces provide stronger electronic interaction and shorter transfer distances. Tan's group synthesized NH_2_–UiO‐66@TpBD‐COF core–shell structure in which the shell thickness determined the effective transfer length [115]. Variation of shell thickness altered carrier exchange rates while preserving structural integrity. Zhou and co‐workers later constructed an interpenetrated MOF@COF hybrid through direct C–C linkage, producing a chemically continuous interface with low transfer resistance (Figure 6) [116]. Although strong coupling improves charge‐separation efficiency, excessive hole transfer to oxidation sites can shorten the lifetime of partially oxidized intermediates unless accompanied by simultaneous microenvironmental stabilization. The main implication is that interfacial coupling should be treated as a flux regulator rather than a parameter to be maximized. Insufficient coupling suppresses activation, whereas overly rapid carrier exchange amplifies radical propagation. Selective CH_4_ photooxidation requires interfacial transport conditions that synchronize carrier flux with intermediate stabilization and diffusion length. Quantitative correlations among interfacial transport dynamics, oxidative flux, and CH_4_ product distribution remain scarce. Time‐resolved operando studies capable of mapping carrier density together with oxygenate survival probability will be needed to define this relationship with precision.

#### 4.2.2 Charge Transfer Topologies

Charge‐transfer topology determines which photogenerated carriers retain a high redox potential and which are eliminated through interfacial recombination [117, 118]. In CH_4_ transformation, this carrier‐selection process directly governs whether oxidation strength is expressed in a form compatible with selective turnover. C─H bond cleavage requires holes with sufficient oxidative potential, while oxygenates preservation requires that this potential not be transferred indiscriminately. In this sense, charge‐transfer topology defines both the magnitude and localization of oxidative flux.

In type‐II heterojunctions, electrons and holes relax to lower‐energy conduction and valence bands on different components. This spatial separation suppresses recombination but also reduces the redox potential of both carriers [110, 119]. For CH_4_ conversion, attenuated hole potential may prolong carrier lifetime yet render C─H activation kinetically inaccessible. Type‐II architecture can therefore favor milder oxidation chemistry, but they may lack the energetic driving force necessary for efficient CH_4_ activation. Z‐scheme and S‐scheme junctions operate according to a distinct carrier‐selection principle [120]. Low‐energy electrons and holes recombine at the interface, while highly oxidizing holes and strongly reducing electrons are retained on separate components [117]. Experimental studies in organic semiconductor heterostructures have provided spectroscopic evidence for this behavior. Ye's group reported vacancy‐enhanced S‐scheme characteristics in g‐C_3_N_4_(NH)/Tp‐Tta COF, supported by Fermi‐level alignment analysis and illumination‐dependent spectroscopy [113]. Li et al. further observed illumination‐induced band bending and directional carrier migration in ZnIn_2_S_4_/g‐C_3_N_4_ composites via in situ XPS and EPR analysis, consistent with S‐scheme transfer [121]. Although these systems were mainly evaluated for CO_2_ reduction, they demonstrate that high‐energy oxidative endpoints can be preserved under steady illumination.

Retention of strong oxidative holes can facilitate the initial C─H activation step in CH_4_ conversion, where the kinetic barrier is substantial. The same high‐energy carriers can also increase the risk of secondary oxidation if spatial confinement and flux moderation are not implemented simultaneously. Z‐scheme and S‐scheme topologies restore thermodynamic strength, but without kinetic and microenvironmental coordination, they may intensify overoxidation rather than improve selectivity. Reticular frameworks further demonstrate that charge‐transfer pathways can be structurally programmed. Le Huec et al. reported a heteroepitaxial MOF‐on‐MOF photocatalyst operating via a Z‐scheme mechanism, supported by spectroscopic and electrochemical evidence [122]. Jiang's group resolved illumination‐induced band bending in a hollow Rh‐COF@COF S‐scheme heterojunction using in situ XPS and Kelvin‐probe measurements [123]. These studies show that charge‐transfer topology can be experimentally established rather than inferred from static band diagrams, enabling more precise correlation between carrier routing and catalytic behavior.

Direct validation of charge‐transfer modes under CH_4_ reaction conditions remains limited. Many assignments rely on surrogate reactions that do not reproduce the activation–preservation asymmetry intrinsic to CH_4_ oxidation. Whether retention of high‐energy holes in S‐scheme systems enhances CH_4_ selectivity or accelerates oxygenate degradation has not been systematically quantified. Time‐resolved operando measurements that correlate band bending, hole potential, and product distribution will be required to resolve this question. Charge‐transfer topology should be evaluated by whether it preserves sufficient oxidative strength for C─H activation while preventing this strength from being transferred indiscriminately to oxygenated products. Selective CH_4_ photooxidation requires carrier‐selection pathways that retain activation capability while spatially and temporally constraining oxidative flux.

#### 4.2.3 Built‐in Electric Field

Built‐in electric fields regulate the spatial distribution of photogenerated carriers under illumination and determine where oxidative equivalents accumulate [109]. In CH_4_ photooxidation, selectivity depends on whether strongly oxidizing holes localize at CH_4_ activation sites while remaining separated from regions where CH_3_OH resides or desorbs. When the activation and preservation domains overlap spatially, secondary oxidation becomes more likely regardless of thermodynamic alignment. Wang et al. constructed a p‐n Na_0.6_CoO_2_/g‐C_3_N_4_ S‐scheme junction and employed in situ XPS and Kelvin probe spectroscopy to monitor illumination‐induced contact potential differences [124]. Although this system was examined in other photocatalytic reactions, it demonstrates that electron‐rich and hole‐rich regions can be dynamically established during operation rather than inferred from static band diagrams. In CH_4_ systems, such dynamic separation may confine oxidative equivalents to defined regions while limiting their interaction with newly formed oxygenates.

Electrostatic steering can be further engineered in MOF‐based junctions through the selection of metal nodes and organic linkers. Shi and co‐workers reported an in situ grown BiOCl/Bi‐MOF heterostructure in which interfacial potential differences were correlated with enhanced carrier separation [125]. Band bending reflected charge redistribution between components and defined the direction of carrier flow. Reticular architecture offers structural flexibility to design these electrostatic gradients with molecular precision. Controlled band bending can concentrate high‐energy holes at designated catalytic motifs while adjacent adsorption domains experience lower oxidative bias. Such spatial differentiation reduces direct overlap between oxidizing centers and desorbing CH_3_OH.

COF‐based systems provide additional evidence of electrostatic regulation. Zhuo et al. identified internal electric fields at the ZnIn_2_S_4_/COF interface that promoted S‐scheme charge transfer under illumination [126]. Photoinduced band bending facilitated recombination of low‐energy carriers while preserving high‐energy electrons and holes on separate components. Although this system was evaluated for H_2_O_2_ production, the same principle is relevant to CH_4_ oxidation. Spatially separated oxidative domains can maintain sufficient potential for C─H activation while reducing the probability that CH_3_OH encounters equivalent hole density immediately after formation.

Electrostatic separation, however, does not automatically guarantee selectivity. Strong internal fields may generate localized carrier accumulation, leading to elevated hole density within confined regions. If adsorption and diffusion are not coordinated, intensified oxidative flux can still promote overoxidation. As such, the effectiveness of built‐in fields depends on coupling electrostatic localization with controlled carrier flux and intermediate residence time. Most experimental demonstrations of internal electric fields have relied on surrogate reactions, and direct operando observation of band bending during CH_4_ conversion remains scarce. Quantitative relationships among field strength, carrier‐density distribution, and CH_4_ product selectivity have yet to be systematically established. Built‐in electric fields contribute to selectivity only when carrier localization is coupled with controlled flux and intermediate residence. Their function should be evaluated as spatial regulation rather than charge separation alone.

### 4.3 Reactive Species Regulation

Reactive species regulation determines the chemical form in which photogenerated redox equivalents are expressed during oxygen activation. In selective CH_4_ photooxidation, reactive species identity acts as a decisive branching point that links CH_4_ activation with product preservation. The same photogenerated carrier flux can generate distinct oxidation trajectories, depending on whether oxygen activation proceeds through diffusive radicals or coordination‐confined intermediates such as surface‐bound *OOH and high‐valent M═O species [112]. Diffusive radicals operate with minimal substrate discrimination and frequently drive runaway oxidation, whereas coordination‐confined intermediates impose geometric and electronic constraints that moderate C─H activation and suppress uncontrolled overoxidation [45]. This distinction is critical for CH_4_ systems, where C─H bond cleavage step demands strong oxidizing equivalents, yet indiscriminate radical flux rapidly erodes selectivity once CH_3_OH is formed. Reactive species regulation should be understood not as a mechanistic label, but as an architectural outcome emerging from the coordination environment, excited‐state manifold, proton management, and spatial charge separation.

#### 4.3.1 Radical‐dominated Regimes

Radical‐dominated regimes are kinetically efficient but structurally indiscriminate. In CN‐based photocatalysis, photogenerated electrons reduce O_2_ to superoxide, which subsequently undergoes proton‐coupled transformations, leading to peroxide formation and subsequent O─O bond cleavage to generate ^•^OH [127]. When homolytic scission proceeds unchecked, freely diffusing radicals dominate the oxidative manifold and extend beyond the catalyst surface into the diffusion layer [128, 129]. Under such conditions, CH_4_ activation and CH_3_OH oxidation become difficult to decouple as radical propagation is not spatially confined. Structural modification can partially redirect this pathway. Wang's group demonstrated that disruption of intrinsic hydrogen‐bond networks in CN exposes additional proton‐accepting sites, accelerates PCET and stabilizes surface‐bound *OOH intermediates [130]. Operando measurements revealed suppression of O─O bond homolysis together with enhanced two‐electron oxygen reduction. Constraining O─O cleavage reduces the flux of freely diffusing ^•^OH and shifts the oxidative field toward the catalyst surface. For CH_4_ conversion, this contraction of the oxidative field is critical as it limits the probability that CH_3_OH encounters diffusive radicals immediately after formation.

**FIGURE 7:**
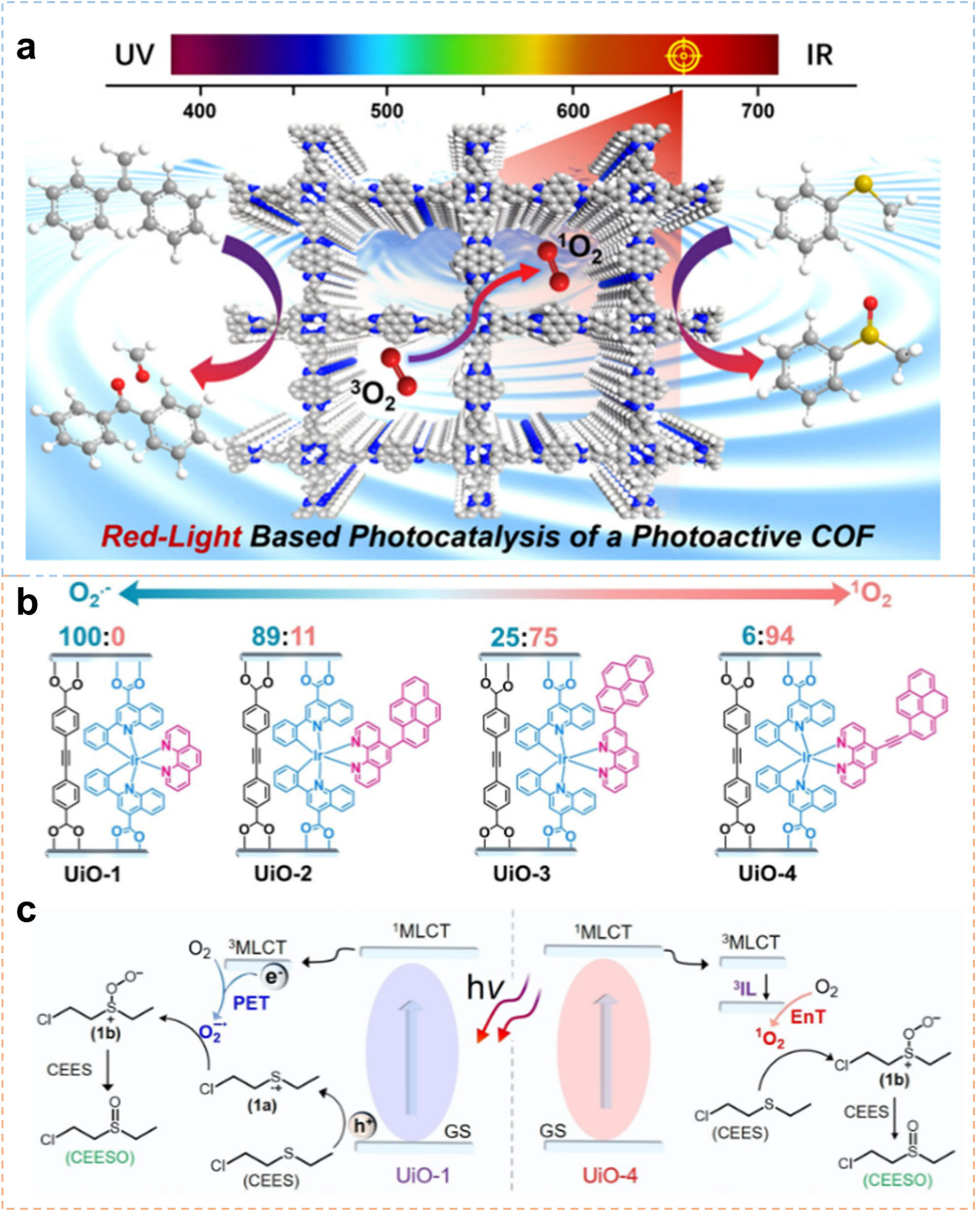
(a) Proposed mechanism for photocatalytic cleavage of olefins (left) and photooxidation of thioanisoles (right). Reproduced with permission [132]. Copyright 2024, American Chemical Society. (b) Structure of photosensitizing MOFs of UiO‐1–UiO‐4 and the ratio of O_2_^•−^ and ^1^O_2_ generation from UiO‐1 to UiO‐4. (c) Mechanism of photocatalytic molecular oxygen activation over UiO‐1 and UiO‐4. EnT: energy transfer. PET: photoinduced electron transfer. Reproduced with permission [139]. Copyright 2025, Wiley‐VCH.

Coordination control provides a related route. Atomically dispersed Cu sites anchored on CN favor coordination‐bound hydroperoxo intermediates rather than freely diffusing radicals [131]. Spin‐trapping EPR and scavenger experiments indicated diminished ^•^OH signatures and enhanced surface‐associated oxygen species. A defined coordination sphere restricts radical escape and decreases secondary oxidation in model reactions. Whether CH_4_ activation remains feasible under such conditions depends on the effective oxidation potential of the bound intermediate. Excess stabilization lowers the accessible redox strength and may render C─H cleavage kinetically limited, even if radical overoxidation is suppressed. Excited‐state structure can also influence radical generation. Niu et al. reported a porphyrinic COF (PyPor‐COF) that selectively generates ^1^O_2_ through energy transfer from long‐lived triplet excitons [132]. Spectroscopic characterization identified ^1^O_2_ as the dominant oxidant. Energy‐transfer pathways circumvent direct electron‐transfer radical chains and can moderate overoxidation in many substrates (Figure 7a). However, ^1^O_2_ exhibits comparatively limited intrinsic reactivity toward CH_4_ under mild conditions. This central lesson suggests that suppressing radical formation alone is insufficient. The remaining oxidant must retain enough driving force for CH_4_ activation while limiting spatial propagation.

#### 4.3.2 Metal‐oxo/Metal–(Hydro)Peroxo Intermediates

Metal‐centered oxygen intermediates confine oxidative chemistry within discrete coordination environments. Species such as M─OOH, M─OO, M─O, and high‐valent M═O operate inside a first coordination sphere rather than propagating through unrestricted diffusion. Hydrogen abstraction occurs at defined metal sites, and oxidative strength is expressed locally. In CH_4_ photooxidation, this spatial confinement reduces overlap between the initial C─H activation step and subsequent CH_3_OH exposure to high‐energy oxidants. Whereas radical pathways expand the oxidative field into the surrounding medium, coordination‐bound intermediates restrict it to geometrically defined pockets.

Organic semiconductor frameworks offer anchoring motifs capable of stabilizing such intermediates under illumination. Nitrogen‐rich CN lattices provide coordination sites for isolated metal atoms, enabling oxygen activation to proceed through surface‐bound intermediates rather than diffusive radicals. Robert's group investigated an Ag single‐atom catalyst on CN (Ag_1_@CN_x_) and proposed a mechanism involving coordination‐bound oxygen species instead of free radical propagation [133]. In this configuration, oxygen activation proceeds via a localized sequence of M─OO and M─O intermediates, with reactivity governed by metal–ligand electronic structure and PCET kinetics. For CH_4_ activation, confinement within a defined coordination sphere can facilitate controlled hydrogen abstraction while limiting secondary radical diffusion. However, accumulation of high‐valent M═O species without synchronized proton transfer may promote rebound‐type processes that accelerate CH_3_OH oxidation. Coordination confinement reduces spatial spread of oxidation but does not eliminate the need for kinetic balance.

Reticular MOF systems extend confinement through periodic metal nodes with uniform geometry. Gao and co‐workers reported a defect‐engineered MOF photocatalyst in which oxygen vacancies promoted end‐on O_2_ adsorption and stabilization of surface‐bound *OOH intermediates along a two‐electron pathway [134]. By favoring associative oxygen activation and suppressing O─O bond homolysis, oxidative chemistry remained restricted to node environments. CH_4_ activation under such conditions occurs at well‐defined sites, yet preservation requires that CH_3_OH desorb or diffuse away before repeatedly encountering the same high‐valent center. Site isolation narrows the oxidative domain but does not automatically prevent overoxidation if product residence time is prolonged. Metal COFs integrate coordination motifs into π‐conjugated frameworks, combining electronic transport with spatial confinement. Shi and co‐workers demonstrated cobalt‐porphyrin‐embedded COFs that enable structured oxygen activation within ordered coordination environments [135]. Carriers migrate through the extended π‐network, whereas oxidative equivalents are localized at porphyrinic sites. For CH_4_ conversion, such architectures can stabilize high‐valent metal–oxo species while maintaining separation from bulk adsorption regions. Nonetheless, porphyrinic centers often possess substantial intrinsic oxidation potentials. Without electronic moderation through ligand design or surrounding framework polarization, excessive oxidative strength may compress the activation–preservation interval and accelerate secondary oxidation.

Cocatalysts represent another critical implementation strategy for constructing coordination‐confined oxygen activation pathways on organic photocatalysts. Rather than functioning only as activity promoters, cocatalysts define local sites where oxidant activation, charge accumulation, and C─H activation are coupled. Atomically dispersed metal centers, multinuclear motifs, or nanoscale clusters anchored on CN, MOFs, and COFs can serve as localized coordination domains for oxygen or peroxide activation, favoring the formation of surface‐bound M─OOH, M─O, or high‐valent M═O species instead of freely diffusing radicals [136]. Shi's group reported Fe single‐atom‐incorporated MOFs for photocatalytic CH_4_ oxidation, where isolated Fe sites provided localized coordination environments for oxygen activation and C─H functionalization [137]. In this system, the incorporation of Fe single atoms into the MOF scaffold enhanced selective CH_4_ oxidation by defining discrete reactive centers rather than relying on unconfined radical chemistry. Zhao and co‐workers developed MOF‐derived heterojunction photocatalysts with precise cocatalyst placement, demonstrating that the spatial distribution of cocatalysts regulates interfacial charge separation and O_2_ activation [138]. This finding highlights that cocatalyst loading affects not only the number of active sites but also the delivery of photogenerated carriers to CH_4_ activation domains.

Furthermore, Ning's group constructed an atomically tailored reticular gas‐solid interface, in which single‐atom cocatalysts were integrated with gas transport pathways to couple in situ oxidant generation, CH_4_ diffusion, and CH_3_OH desorption within a structured architecture [77]. These investigations show that cocatalysts influence pathway selection through three coupled functions. First, they regulate the identity of oxygen intermediates by favoring site‐bound M─OOH, M─O, or M═O species over freely diffusing radicals. Second, they act as local charge‐accumulation centers that improve photogenerated carrier utilization for oxygen activation and subsequent C─H activation. Third, they modify the spatial relationship between CH_4_ activation and oxygenate preservation by confining reactive intermediates near defined sites while limiting non‐selective radical propagation. As a consequence, cocatalyst loading contributes to selectivity not simply by increasing activity, but by integrating oxygen activation mode, charge routing, and intermediates confinement within a single local environment.

The key requirement for metal‐centered pathways is not merely stabilizing M─OOH or M═O intermediates but coordinating their lifetime with product escape from the same coordination domain. C─H activation occurs within coordination pockets, and oxygenated products exit those pockets before further oxidation proceeds. Direct operando identification of high‐valent M═O species during CH_4_ photoconversion remains scarce, and a quantitative correlation between coordination‐sphere intermediate lifetime and CH_3_OH yield has not yet been established. Establishing this relationship is essential for translating coordination confinement into predictable CH_4_ selectivity.

#### 4.3.3 Spin‐State Regulation

Spin‐state regulation dictates reactive species identity by biasing oxygen activation toward either electron‐transfer‐driven radical formation or energy‐transfer‐mediated ^1^O_2_ production [140]. These routes differ in both oxidation strength and spatial propagation. Electron‐transfer processes typically produce highly reactive radical species capable of initiating C─H bond cleavage, yet such species diffuse beyond the coordination sphere and often propagate secondary oxidation. Energy‐transfer pathways mediated by triplet excitons impose spin and geometric constraints that moderate reactivity and limit radical chains [141, 142]. However, ^1^O_2_ exhibits comparatively weak intrinsic reactivity toward CH_4_ under mild conditions, which restricts its ability to accomplish primary C─H activation independently.

In CN systems, modifications to the excited‐state manifold shift the balance between these pathways. Zhao et al. reported carbonylated CN exhibiting photo‐switchable ROS generation [143]. Transient absorption spectroscopy revealed altered relaxation channels, enabling reversible switching between radical‐ and ^1^O_2_‐dominated regimes. Redistribution of excited‐state decay pathways modified ROS identity without altering the underlying lattice. An increased triplet population suppressed radical flux and reduced diffusive oxidation. For CH_4_ conversion, such moderation is advantageous only if the resulting oxidant retains sufficient thermodynamic strength for the first hydrogen abstraction step. Microstructural order further influences spin accessibility. Engineering of PCN frameworks can stabilize triplet states and modify exciton decay dynamics, thereby enhancing ^1^O_2_ generation [144]. Triplet yield reflects electronic coupling and lattice organization. Constraining radical formation narrows the spatial extent of the oxidative field, yet C─H cleavage may remain kinetically demanding unless additional activation motifs, such as metal–oxo centers, operate in parallel.

**FIGURE 8:**
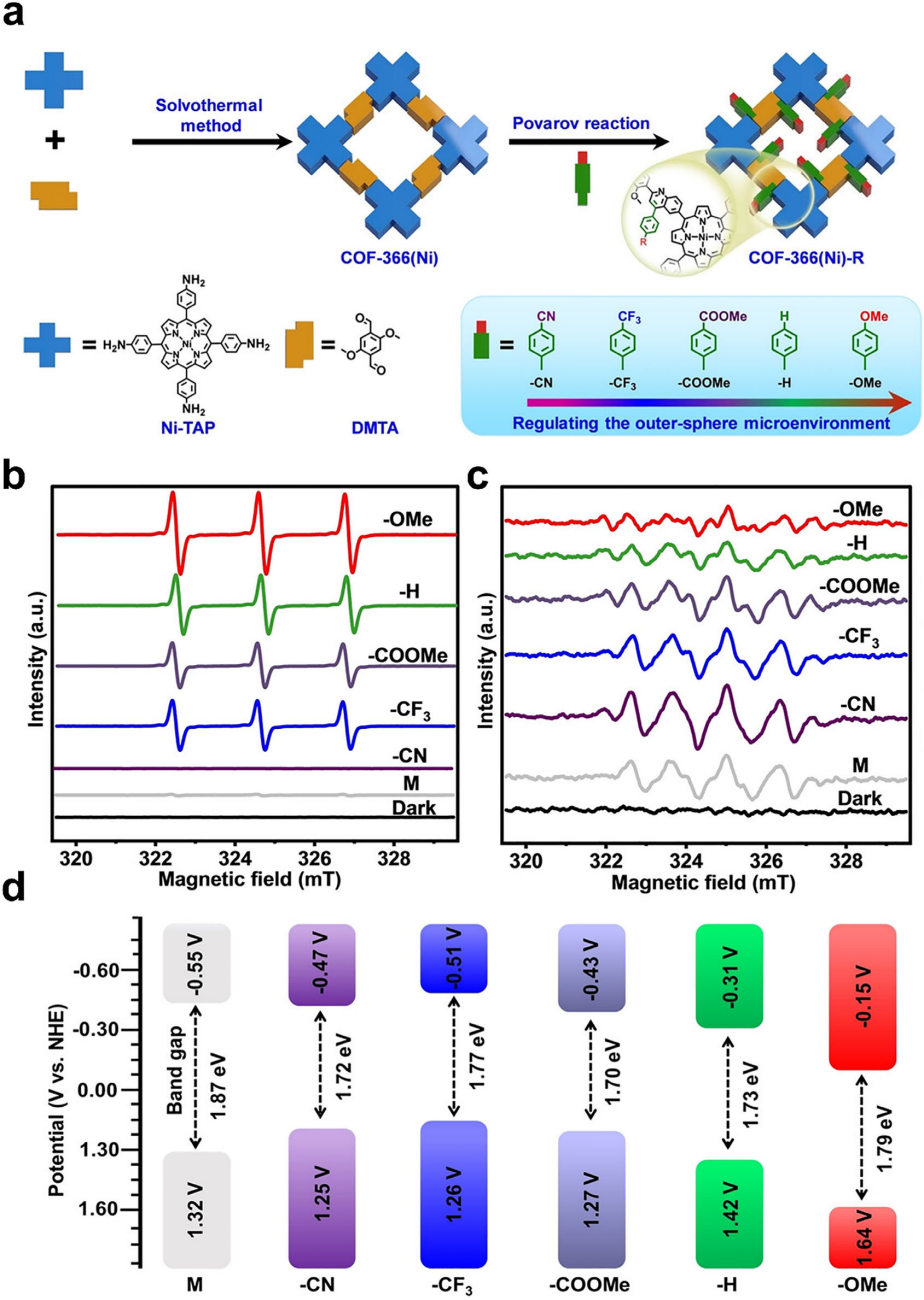
(a) Schematic representation of the outer‐sphere microenvironment modulation of Ni sites in COF‐366(Ni)‐R (‐R = ‐CN, ‐CF_3_, ‐COOMe, ‐H, and ‐OMe). EPR spectra of COF‐366(Ni) and COF‐366(Ni)‐R in the presence of (b) TEMP and (c) DMPO. (d) Band gaps of COF‐366(Ni) and COF‐366(Ni)‐R. Reproduced with permission [145]. Copyright 2023, Wiley‐VCH.

In MOF photosensitizers, spin‐state control can be tuned by the metal node identity and ligand‐to‐metal charge‐transfer characteristics. Ma et al. analyzed the competition between electron‐transfer and energy‐transfer pathways in MOF‐based systems and showed that localized triplet excitons favor ^1^O_2_ formation, whereas charge‐transfer states promote superoxide generation (Figure 7b,c) [139]. Variation of metal identity alters spin‐orbit coupling and excited‐state lifetime, thereby adjusting the ^1^O_2_ to superoxide ratio. For CH_4_ oxidation, increasing the contribution of energy‐transfer channels may reduce formation of highly reactive radicals, although complete suppression of electron‐transfer pathways could compromise C─H activation. Porphyrinic COFs provide additional evidence that confinement affects triplet persistence. Jiang's group showed that systematic modulation of outer‐sphere microenvironment in COF‐366(Ni)‐R frameworks suppressed charge‐transfer quenching and enhanced ^1^O_2_ sensitization within ordered frameworks (Figure 8a) [145]. EPR measurements demonstrated that TEMP‐derived signals assigned to ^1^O_2_ are enhanced under electron‐donating environments, while DMPO‐detected radical signals show an inverse trend (Figure 8b,c). This inverse correlation reflects a shift from electron‐transfer to energy‐transfer pathways, providing direct evidence that spin‐state regulation governs reactive species identity. This behavior is further supported by the corresponding variation in band‐edge positions (Figure 8d), which modulates the thermodynamic feasibility of electron‐transfer reduction of O_2_ and thereby biases oxygen activation towards energy‐transfer pathways.

Spin‐state regulation thus influences both the origin and the diffusion behavior of reactive oxygen species. Selectivity benefits when radical propagation is curtailed and oxidative chemistry are confined within controlled lifetimes and spatial domains. Yet CH_4_ activation imposes a stringent requirement that moderated oxidants retain sufficient strength to cleave the first C─H bond. The balance between excitation dynamics and activation capability remains system‐specific. Direct operando correlations among triplet lifetime, reactive‐species distribution, and CH_4_ product selectivity remain limited, and establishing this linkage is essential for rational excited‐state design. The design target is not radical suppression alone, but activation‐capable moderation of oxidative species.

Overall, reactive species regulation determines how oxidative equivalents are chemically expressed and spatially propagated. Diffusive radicals offer strong activation capability but poor molecular discrimination, while coordination‐confined and spin‐regulated routes restrict oxidative spread. Therefore, selective CH_4_ photooxidation requires reactive species with appropriate strength, lifetime, and locality. These constraints lead directly to microenvironmental regulation, where confinement, adsorption, and proton organization determine whether oxygenated intermediates escape or remain exposed to further oxidation.

### 4.4 Microenvironment Regulation

Microenvironmental regulation provides the spatial and temporal control needed to translate reactive species selectivity into product preservation. It defines how oxidizing equivalents encounter partially oxidized intermediates during turnover. In CH_4_ photooxidation, selectivity hinges on whether C─H activation terminates at oxygenates or proceeds toward deep mineralization. CH_3_OH is intrinsically more reactive than CH_4_, and its survival depends on exposure time within strongly oxidizing domains. The governing parameter is not adsorption strength alone, but the residence time of intermediates within oxidative regions relative to their desorption and diffusion rates.

In porous organic semiconductors, confinement, polarity, hydrogen‐bond organization, and interfacial solvation shape this residence time [57, 146, 147, 148]. These parameters operate downstream of thermodynamic alignment and reactive‐species identity. Even when oxidative strength is properly positioned and radical propagation is suppressed, microenvironmental control determines whether oxidative equivalents remain localized or whether intermediates are repeatedly exposed to them. Selectivity depends on whether product evacuation outpaces oxidative recurrence.

#### 4.4.1 Confinement and Diffusion

Confinement governs CH_4_ selectivity by controlling how long partially oxidized intermediates remain within oxidative domains. CH_4_ activation and CH_3_OH oxidation often occur within overlapping spatial regions. Selectivity improves only when CH_3_OH diffusion away from high‐valent centers proceeds faster than radical propagation or surface rebound reactions [149]. As the intermediate residence time approaches or exceeds the recurrence time of oxidative species, secondary oxidation becomes increasingly likely [150]. Pore geometry and defect distribution establish this balance. Increasing porosity enhances molecular transport and reduces local accumulation of oxidative species near activation sites. Mesoporous CN architectures with hierarchical channels facilitate diffusion and lower the steady‐state concentration of oxidants near activation sites [151, 152]. Under controlled conditions, improved transport limits repeated exposure of CH_3_OH to high‐energy oxidants. Spectroscopic and transient analyses indicate that vacancy density affects carrier lifetime and the local charge distribution, which in turn influence intermediate stabilization [153]. However, excessive defect concentration may trap oxygenated species near active sites and prolong their exposure to oxidative flux. Confinement acts as a kinetic regulator only when desorption dynamics and carrier distribution are simultaneously balanced.

**FIGURE 9:**
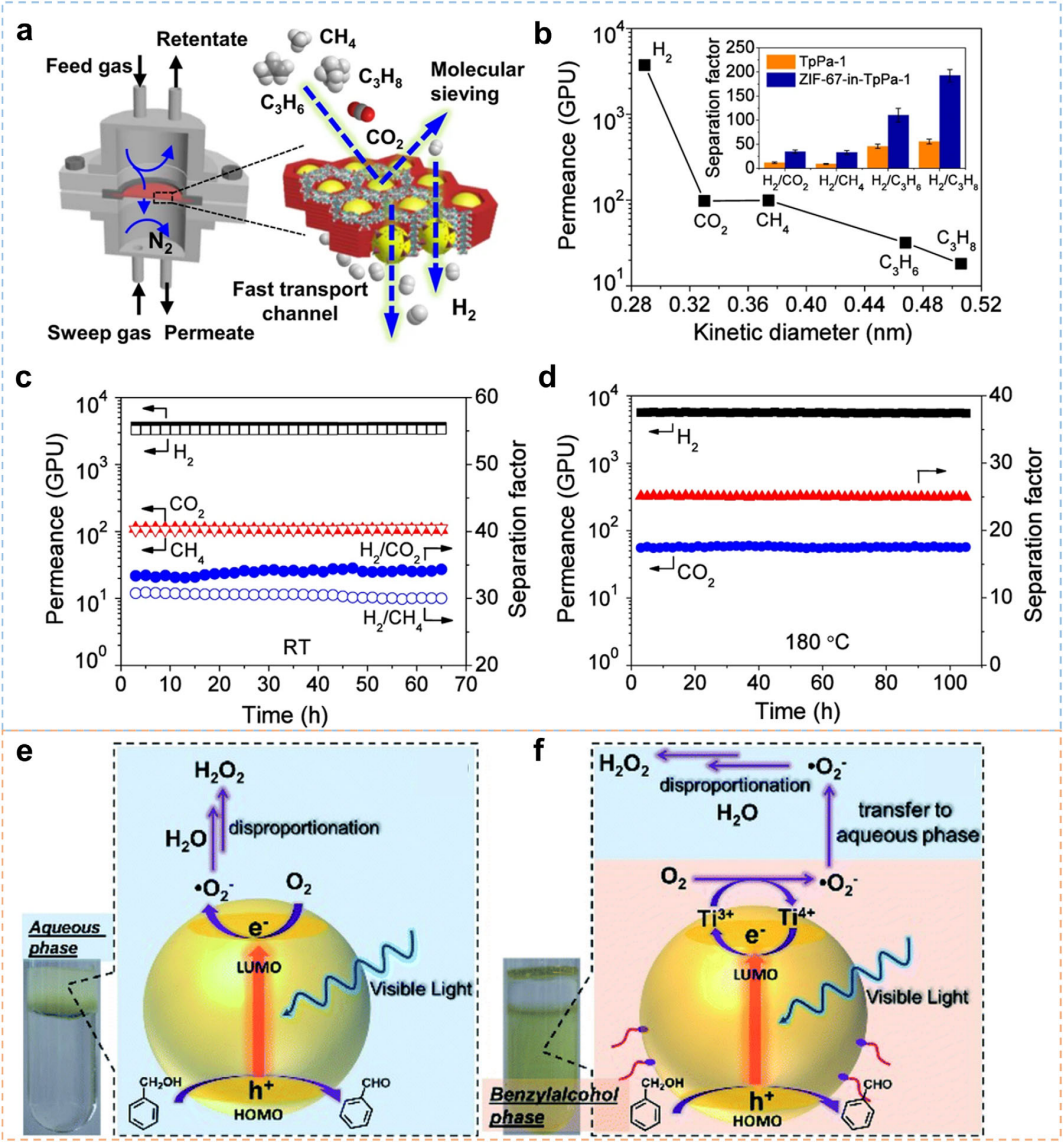
(a) Home‐made gas‐permeation module and schematic illustrating gas transport through ZIF‐67‐in‐TpPa‐1 membrane. (b) Single‐gas permeances of ZIF‐67‐in‐TpPa‐1 membrane as a function of kinetic diameter of permeating molecules at 298 K and 1 bar. Inset shows the mixed‐gas‐separation factor of ZIF‐67‐in‐TpPa‐1 membrane and TpPa‐1 membrane for H_2_ over other gases. Long‐term tests of ZIF‐67‐in‐TpPa‐1 membrane for (c) equimolar gas mixture of H_2_/CO_2_ and H_2_/CH_4_ at 298 K and 1 bar, and (d) equimolar gas mixture of H_2_/CO_2_ at 453 K (180°C) and 1 bar. Reproduced with permission [156]. Copyright 2021, Springer Nature. Proposed mechanism for photocatalytic H_2_O_2_ production using (e) Zr100‐MOF and (f) OPA/Zr92.5Ti7.5‐MOF. Reproduced with permission [159]. Copyright 2021, Royal Society of Chemistry.

Reticular MOFs provide crystallographically defined diffusion pathways whose aperture and connectivity can be tuned with molecular precision. Narrow channels can enrich CH_4_ at metal nodes and increase local concentration, potentially lowering the apparent activation barrier. Meanwhile, restricted apertures may retard CH_3_OH desorption and extend its residence within oxidative pockets. Subnanometer channel engineering has revealed that transport in MOFs deviates from simple Fickian diffusion and becomes sensitive to steric and electrostatic interactions (Figure 9a–d) [154, 155, 156]. Although many of these studies address ion transport or membrane separation, the same physical principles apply to catalytic systems in which diffusion and surface reaction compete on comparable time scales. Therefore, channel geometry influences not only substrate delivery but also the temporal separation between CH_4_ activation and product oxidation. COFs provide periodic one‐ or two‐dimensional channels with tunable diameter and wall polarity. Variation in pore size alters encounter frequency between adsorbates and reactive sites [157]. Hierarchically porous COFs integrating micro‐ and mesopores have demonstrated decoupling of mass transport from catalytic turnover in other photocatalytic reactions [158]. Such hierarchical organization may allow CH_4_ enrichment within microporous domains while enabling rapid CH_3_OH evacuation through larger transport channels, thereby shortening oxygenate residence time in high‐energy environments.

The key design rule is that confinement improves selectivity only when it shortens oxygenate residence relative to oxidative recurrence. Quantitative relationships among pore aperture, diffusion coefficient, carrier density, and product distribution remain largely unexplored under CH_4_ photooxidation conditions. Time‐resolved operando measurements capable of mapping concentration gradients together with product evolution are required to establish direct confinement‐selectivity coupling.

#### 4.4.2 Adsorption Geometry

Adsorption geometry governs which species occupy oxidative domains and how long they remain there [160]. In CH_4_ photooxidation, activation and secondary oxidation often occur at adjacent or identical surface sites [149]. Selectivity depends not only on adsorption strength, but also on differential binding geometries and residence times. CH_4_ activation requires transient interaction with oxidizing centers, whereas CH_3_OH preservation requires that the oxygenate either bind weakly or reorient away from high‐flux domains before further oxidation proceeds.

Surface polarity and local electrostatics reshape adsorption configuration by altering molecular orientation and intermediate stabilization. Introduction of polar functional groups modifies how oxygen species bind and react at catalytic sites. Sulfonic‐acid functionalization enhances hydrophilicity and proton transport while altering the surface charge distribution, thereby promoting associative O_2_ activation via stabilized *OOH intermediates [161]. Defect‐associated frustrated Lewis pairs further bias adsorption geometry and intermediate stabilization, favoring controlled oxygen activation rather than indiscriminate radical formation [162]. These modifications regulate how oxidants are positioned relative to adsorption pockets. However, increasing polarity may simultaneously strengthen CH_3_OH adsorption at the same sites, prolonging its residence within oxidative domains. Functionalization must therefore generate adsorption asymmetry between CH_4_ and CH_3_OH rather than uniformly enhancing affinity.

**FIGURE 10:**
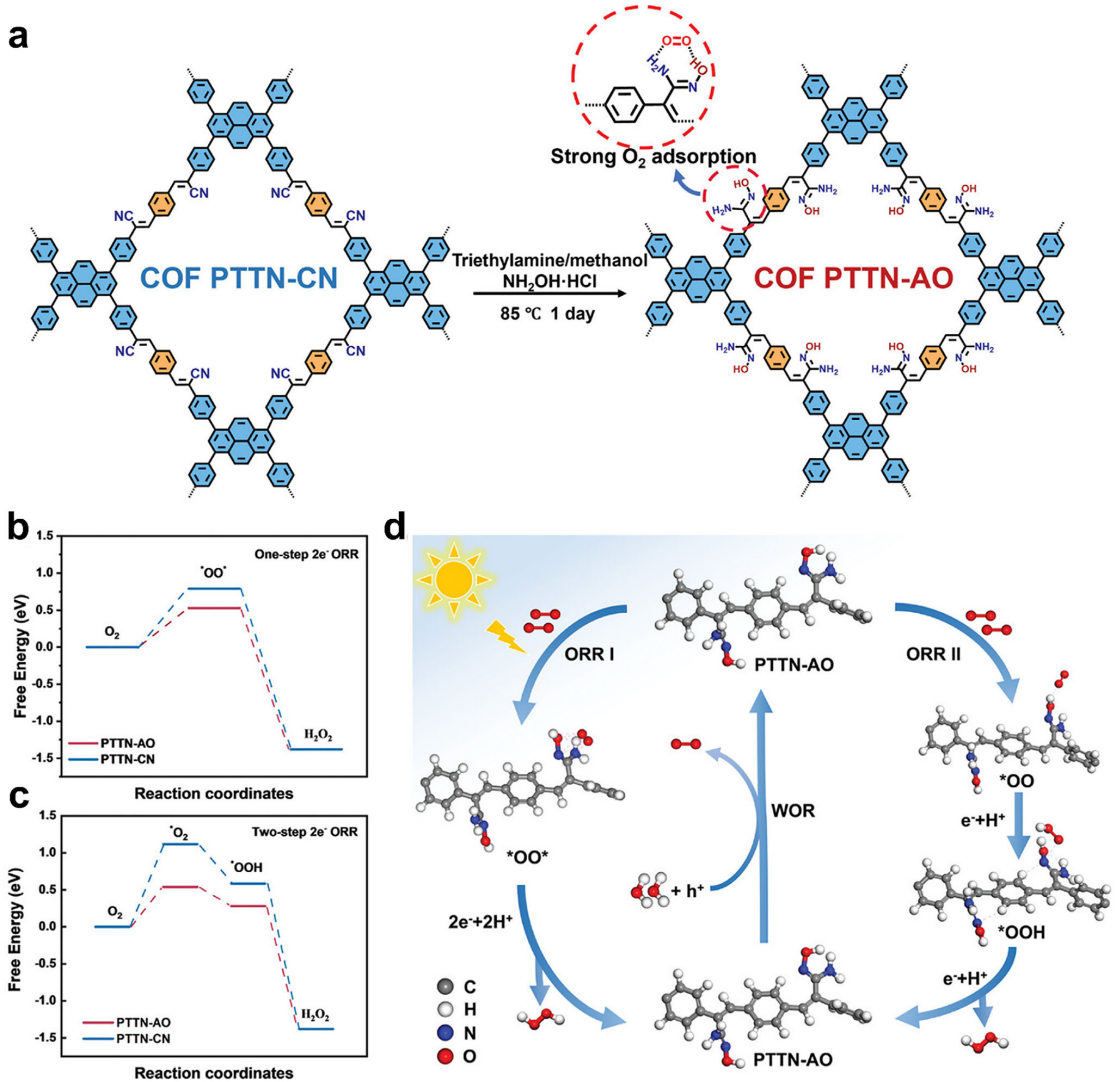
(a) Synthesis of PTTN‐AO. (b) Free‐energy diagrams for one‐step 2e^−^ ORR to H_2_O_2_ on PTTN‐CN and PTTN‐AO. (c) Free‐energy diagrams for two‐step 1e^−^ ORR to H_2_O_2_ on PTTN‐CN and PTTN‐AO. (d) Proposed reaction mechanism toward H_2_O_2_ production on PTTN‐AO. Reproduced with permission [166]. Copyright 2025, Wiley‐VCH.

Control over pore‐wall polarity offers additional leverage. Modification of linker environments in Zr‐based MOFs alters local electric fields and proton‐donor configurations at metal nodes [163]. Hydrophobic functionalization has been shown to spatially separate peroxide formation from catalytic centers in biphasic systems [159, 164]. Partial hydrophobicity within pores can facilitate CH_3_OH desorption into the surrounding phase, thereby shortening oxygenate residence time (Figure 9e,f). Excessive hydrophobicity, in contrast, restricts water accessibility and disrupts PCET necessary for controlled oxygen activation. Adsorption geometry must operate within a narrow balance in which oxygen activation remains efficient while oxygenate retention is minimized. Periodic functional group arrangement in COFs enables further refinement of adsorption orientation. Electron‐withdrawing cyano substitution modifies local electrostatic potential and stabilizes associative oxygen intermediates relative to superoxide pathways [165]. Post‐synthetic amidoxime functionalization introduces directional hydrogen‐bond interactions that lower barriers for two‐electron oxygen reduction (Figure 10) [166]. These structural modifications influence the positioning of oxygen and intermediates within confined pores. For CH_4_ systems, the critical question is whether such tuning creates differential binding lifetimes between CH_4_ and CH_3_OH at identical coordination pockets. Stabilization of activation‐relevant intermediates enhances activity only when oxygenated products experience shorter‐lived interactions at those same sites.

Adsorption geometry also acts as a spatial discriminator only when it differentiates the residence times of CH_4_ and its oxygenated products. Selectivity improves when CH_4_ interacts transiently with oxidizing centers while CH_3_OH is displaced, reoriented, or expelled from high‐flux regions. Direct spectroscopic interrogation of CH_4_ and CH_3_OH binding configurations under illumination remains limited, and quantitative comparison of adsorption lifetimes within identical coordination environments remains unavailable. Such measurements are essential for translating adsorption engineering into predictive CH_4_ selectivity.

#### 4.4.3 Hydrogen‐Bond Networks

Hydrogen‐bond organization regulates proton flux within oxidative microdomains and governs PCET dynamics [167, 168]. In CH_4_ transformation, PCET processes control both the formation of high‐valent oxidants and the subsequent fate of oxygenated intermediates. The spatial arrangement of proton donors and acceptors determines whether oxygen activation proceeds through associative pathways that stabilize *OOH intermediates or shifts toward radical‐generating homolysis [169]. Selectivity depends on synchronizing proton transfer with electron flow while preventing radical propagation within the same coordination domain. Reorganization of hydrogen‐bond networks reshapes this balance. Lu and co‐workers demonstrated that disruption of intrinsic hydrogen‐bond motifs in CN alters the distribution of proton‐accepting nitrogen sites and accelerates PCET during oxygen reduction [130]. Stabilization of *OOH intermediates and suppression of ^•^OH formation correlated with modified hydrogen‐bond environments. Zeng et al. further showed that surface grafting of proton‐donating polymers establishes localized proton‐shuttling pathways that facilitate concerted electron‐proton transfer [170]. Enhanced proton mobility changes the chemical trajectory of oxygen activation.

However, excessive proton availability can accelerate peroxide decomposition and increase radical flux. Proton density must be coordinated with carrier transport rather than increased indiscriminately. Structured proton‐relay networks in reticular frameworks provide additional control. Kondo et al. demonstrated that missing‐linker defects in Hf‐based UiO‐66‐NH_2_, combined with isolated Ni single atoms, localize hydrogen‐bond domains and lower PCET barriers during oxygen activation [171]. In situ spectroscopic investigations of semiconductive MOFs further indicate that proton‐transfer microenvironments influence the distribution of reactive species [172]. Proton relays can confine activation‐relevant intermediates within coordination pockets. Yet strong hydrogen‐bond stabilization of CH_3_OH within the same pocket may prolong its residence time and increase the probability of secondary oxidation.

Importantly, proton organization must differentiate between activation intermediates and oxygenated products. Periodic incorporation of acid–base motifs in COFs enables directional proton transport. Zwitterionic squaric‐acid‐based COFs introduce defined proton‐transfer channels that stabilize associative oxygen intermediates [103]. Wettability tuning in COF membranes reorganizes interfacial water structure and modifies PCET kinetics [173]. These findings indicate that hydrogen‐bond networks determine whether oxygen activation proceeds via confined associative routes or transitions to radical pathways. For CH_4_ conversion, internal proton‐shuttling architectures should promote synchronous PCET during C─H activation while avoiding proton‐assisted radical amplification, which erodes selectivity.

From a dynamic perspective, hydrogen‐bond architecture also controls the temporal coupling between proton transfer and oxidative chemistry. Its selectivity benefit arises only when proton transfer supports activation without prolonging oxygenate residence or amplifying radical formation. Direct operando mapping of proton dynamics during CH_4_ photoconversion remains scarce, and the quantitative correlation between proton‐relay organization and oxygenate yield has not yet been established.

Taken together, microenvironmental regulation provides the spatial and temporal component of activation‐preservation decoupling. Pore architecture, adsorption geometry, hydrogen‐bond organization, and polarity gradients determine whether CH_4_, oxidants, and oxygenated products occupy the same oxidative domains or are separated during turnover. Selectivity improves when CH_4_ activation occurs within confined oxidative regions, while oxygenated products are rapidly displaced or transported away before secondary oxidation proceeds.

Therefore, selective CH_4_ photoconversion is governed by four interdependent regulatory dimensions. Thermodynamic regulation defines the oxidation window, kinetic regulation determines carrier transport, reactive species regulation establishes the oxygen‐activation manifold, and microenvironmental regulation controls intermediate residence. These dimensions must be synchronized within a unified architecture, providing the basis for the architectural evolution discussed below.

## 5 Architectural Evolution Toward Selective CH_4_ Oxidation

Selective CH_4_ photooxidation requires concurrent control of oxidative strength, carrier transfer, reactive species identity, and intermediate residence. Although these variables can be discussed individually, their catalytic consequences are ultimately determined by how they are integrated within a specific framework architecture. Therefore, the focus shifts from isolated regulatory dimensions to the structural arrangements that couple CH_4_ activation with oxygenate preservation.

Organic photocatalyst systems can be organized into radical‐dominated, coordination‐confined, and spatially partitioned architectures according to the extent of structural control over this activation–preservation relationship. Radical‐dominated systems rely primarily on kinetic balancing of reactive flux. Coordination‐confined systems localize oxygen activation within defined sites. Spatially partitioned systems further separate oxidant generation, substrate activation, and product desorption in space and time. This architectural progression provides a basis for comparing catalyst systems and identifying the structural features required for selective CH_4_ photooxidation.

### 5.1 Radical‐Dominated Architectures

**FIGURE 11:**
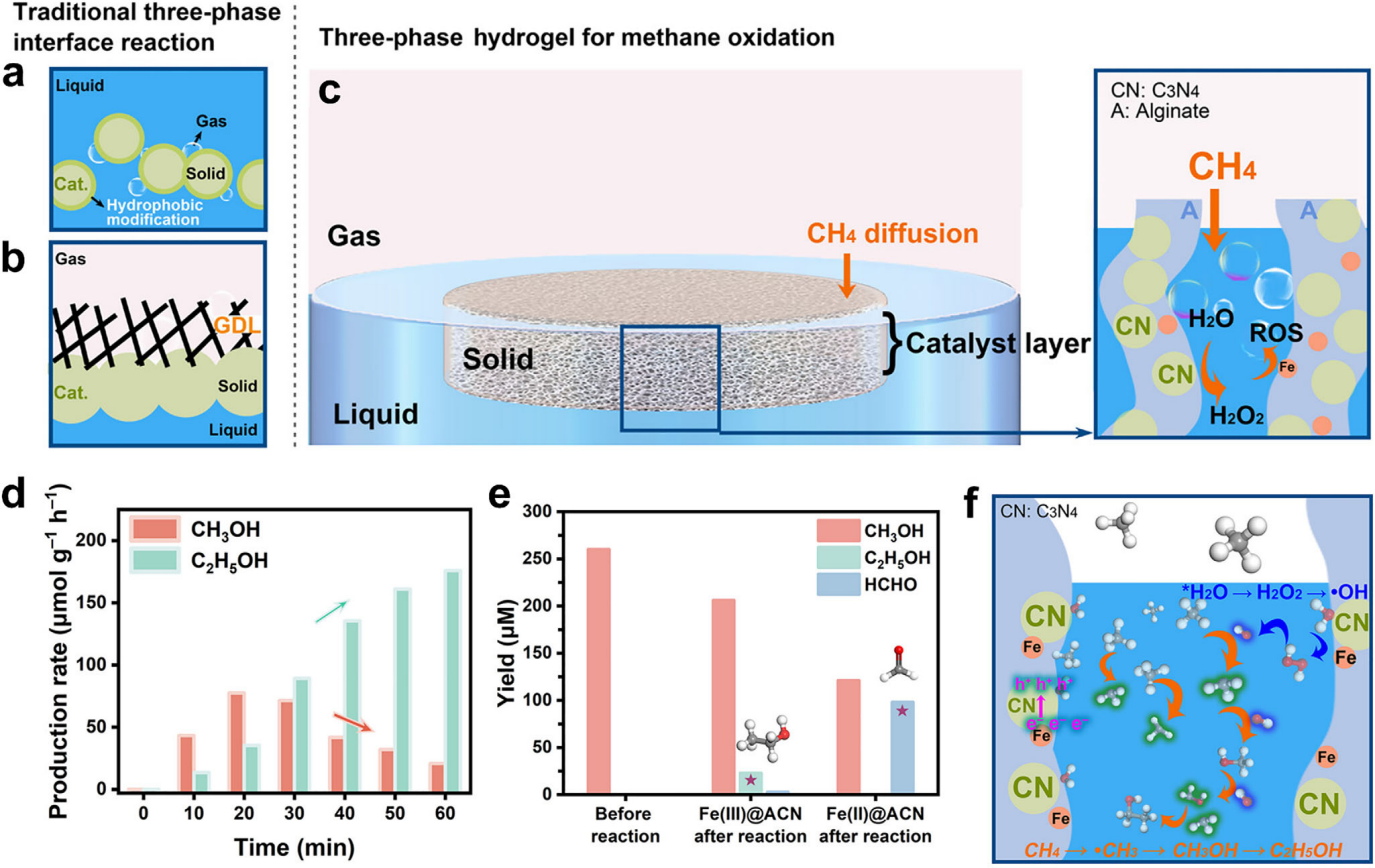
(a) Surface hydrophobic modification of the suspended catalyst or (b) introducing a film with a hydrophobic gas diffusion layer (GDL) at the gas‐liquid interface to reduce gas diffusion distance toward the solid and maintain a suitable wettability. (c) Hydrophilic floating hydrogel for photocatalytic methane oxidation at the three‐phase interface. (d) Reaction time of CH_3_OH and C_2_H_5_OH evolution in Fe(III)@ACN system. (e) CH_3_OH conversion catalyzed by Fe(III)@ACN and Fe(II)@ACN with 0.26 mM CH_3_OH in H_2_O under Ar atmosphere (reaction time: 0.5 h). (f) Schematic illustration of the three‐phase Fe(III)@ACN hydrogel film for the conversion of CH_4_ to C_2_H_5_OH. Reproduced with permission [179]. Copyright 2024, American Chemical Society.

Radical‐dominated architectures represent the earliest and least structurally encoded regime for photocatalytic CH_4_ conversion [174, 175, 176, 177, 178]. In these systems, CH_4_ activation and oxygenate exposure occur within largely overlapping oxidative domains, and selectivity is mainly governed by kinetic balancing of radical flux, substrate accessibility, and product residence. The main limitation is not insufficient oxidative ability, but the absence of intrinsic spatial discrimination between activation and preservation. Thus, high selectivity is confined to a narrow operating window in which radical generation, substrate transfer, and oxygenate desorption remain finely balanced. A representative example is the three‐phase floating‐film strategy, which employs a hydrophilic Fe(III)‐crosslinked macroporous alginate hydrogel encapsulating CN (Fe(III)@ACN) [179]. This architecture improves the accessibility of CH_4_ and H_2_O at the gas‐liquid‐solid interface and balances the local formation and consumption of reactive species, thereby biasing product distribution toward ethanol (Figure 11). The high alcohol selectivity demonstrates that interfacial mass‐transport engineering can partially compensate for the poor spatial discrimination of radical chemistry.

Nevertheless, activation and preservation remain located within the same oxidative environment, so increased local oxidative flux or prolonged oxygenate residence can still promote secondary oxidation. A related design integrates photothermal and photocatalytic functions in Cu_9_S_5_/Cu‐CCN, where local thermal effects and Cu‐mediated oxygen activation enhance C─H activation and C─C coupling [180]. Although ethanol selectivity and productivity are improved, activation and preservation still occur within overlapping oxidative domains.

These radical‐dominated architectures demonstrate that interfacial engineering and transport modulation can enhance apparent selectivity. However, activation and preservation remain co‐localized, and higher activation rates inevitably increase the probability of oxygenate oxidation. Selectivity is stabilized only within a constrained operating window, rather than being encoded intrinsically through architectural decoupling. This limitation motivates the transition toward regimes in which reactive species identity and spatial confinement are more explicitly programmed.

### 5.2 Coordination‐Confined Oxygen Activation

A higher level of structural control is achieved when oxygen activation is localized within specific coordination environments. In this regime, selectivity is no longer maintained primarily by suppressing radical flux, but by defining where oxidative intermediates are generated and how far they can propagate. Organic frameworks provide metal nodes, isolated single‐atom sites, and porphyrinic coordination centers that confine oxygen activation within spatially restricted domains. Compared with radical‐dominated architectures, coordination‐confined systems encode pathway discrimination into the local structure of the catalyst. Dicopper motifs stabilized on CN illustrate this principle [181]. Dimeric Cu sites anchored within the nitrogen‐rich cavities of C_3_N_4_ establish discrete oxygen‐activation domains, enabling CH_4_ conversion with improved selectivity compared to more diffusive radical systems. The decisive architectural feature is the geometric definition of the active site, which confines oxidative chemistry to local coordination environments rather than allowing reactive species to propagate through the surrounding medium.

**FIGURE 12:**
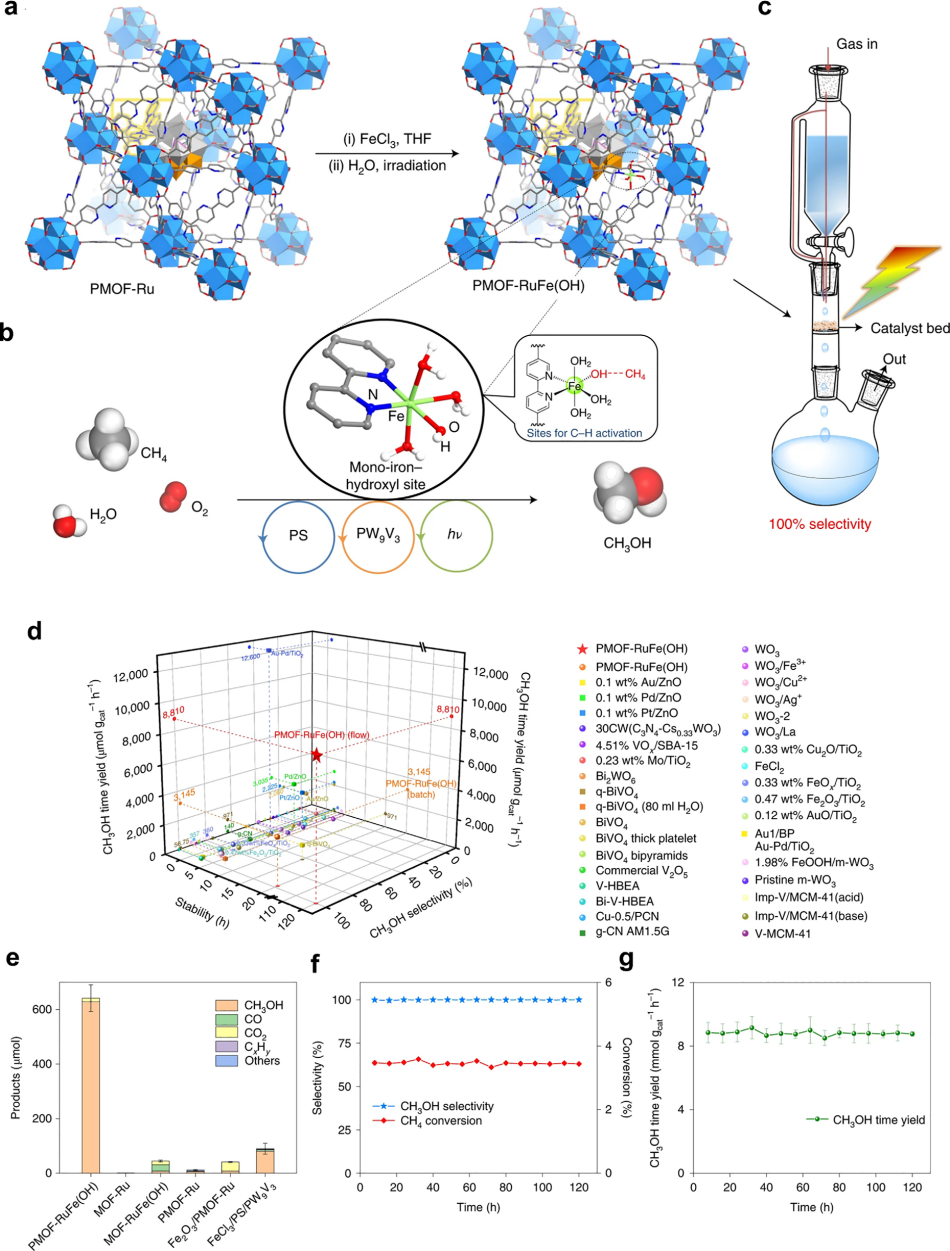
Design and synthesis of PMOF‐RuFe(OH) catalyst and the flow reactor for photo‐oxidation of CH_4_ to CH_3_OH (a–c). (a) View of the structure of PMOF‐RuFe(OH) incorporating mono‐iron hydroxyl, [Ru^II^(bpy)_2_(bpydc)] and [PW_9_V_3_O_40_]^6−^ species within the pore of UiO‐67. All three components are shown in one unit cell to simplify the overall model, and a homogeneous distribution of these components across the entire structure is observed. Labelling scheme: C, grey; O, red; H, white; N, blue; Fe, green; V, orange; W, dark grey; P, purple; Zr_6_ cluster, blue polyhedral; [Ru^II^(bpy)_2_(bpydc)] (PS), gold. (b) Schematic showing synergic catalysis in PMOF‐RuFe(OH) for selective photo‐activation of C─H bond in CH_4_ to CH_3_OH in the presence of oxygen and water. *hν*, irradiation. (c) View of the continuous‐flow apparatus for photocatalytic conversion of CH_4_ to CH_3_OH over PMOF‐RuFe(OH). The wavelength of light used is 400–780 nm. (d) Comparison of the catalytic activity for photo‐oxidation of CH_4_ to CH_3_OH over PMOF‐RuFe(OH) with other photocatalysts. Red stars and orange spheres represent the activity of PMOF‐RuFe(OH) in flow and batch mode, respectively. Other spheres represent the activity at ambient pressure, and cubes represent the activity at high pressure. (e) Comparison of the catalytic activity over various catalysts in batch mode. Reaction conditions: 3 ml water, 20 h visible light, 10 mg catalyst and CH_4_/O_2_ (1 atm). (f) CH_3_OH selectivity and CH_4_ conversion, and (g) time yield for formation of CH_3_OH over a period of 120 h on stream over PMOF‐RuFe(OH) in flow mode. Reproduced with permission [184]. Copyright 2022, Springer Nature.

Single‐atom catalysts further highlight how subtle changes in coordination geometry influence pathway selectivity. Asymmetrically coordinated Co sites on CN and Cu single atom systems with tailored N‐ or O‐coordination spheres regulate O_2_ adsorption and suppress excessive radical generation, leading to improved selectivity of CH_3_OH or HCHO under optimized conditions [182, 183]. These studies indicate that the spatial definition of oxygen activation sites, rather than metal identity alone, governs the transition from radical flux balancing to coordination‐guided pathway control. Coordination‐confined oxygen activation has also been achieved within photoactive MOF lattices. In PMOF‐RuFe(OH), mono‐iron hydroxyl sites, photosensitizing units, and redox mediators are organized within a reticular scaffold, enabling selective CH_4_‐to‐CH_3_OH conversion under visible light (Figure 12) [184]. The framework integrates light absorption, carrier transport, and localized C‐H functionalization within a periodic architecture, demonstrating how coordination confinement can be implemented at the system level.

Despite these advances, activation and preservation remain spatially proximate in coordination‐confined systems. CH_3_OH is often generated near the same coordination sites responsible for oxygen activation, so prolonged residence or intensified local carrier accumulation can still trigger secondary oxidation. This regime represents a transition from kinetic balancing to pathway regulation, but complete architectural synchronization requires additional spatial or temporal separation between oxidative domains and product residence zones.

### 5.3 Spatially Partitioned Architectures

A further stage of architectural evolution is realized when CH_4_ activation, oxidant generation, and oxygenate removal are spatially separated within the catalytic system. Unlike radical‐dominated architectures that rely on flux balancing or coordination‐confined systems that localize oxygen activation, spatially partitioned architectures regulate where each elementary function occurs and how long intermediates remain exposed to oxidative domains. An atomically modified MOF membrane nanoreactor exemplifies this strategy [77]. Single Pd atoms and Fe species are anchored within a reticular scaffold that enables tandem H_2_O_2_ formation and CH_4_ oxidation. The breathable membrane establishes a defined gas‐solid interface that enhances CH_4_ diffusion while restricting uncontrolled interaction between CH_3_OH and ROS. Continuous CH_3_OH production with high selectivity is achieved under mild conditions. In this configuration, oxidant formation, carrier transport, and product evacuation are arranged within a structured interface that confines radical dispersion and shortens oxygenate exposure time. Activation and preservation are not co‐localized but distributed across complementary domains.

**FIGURE 13:**
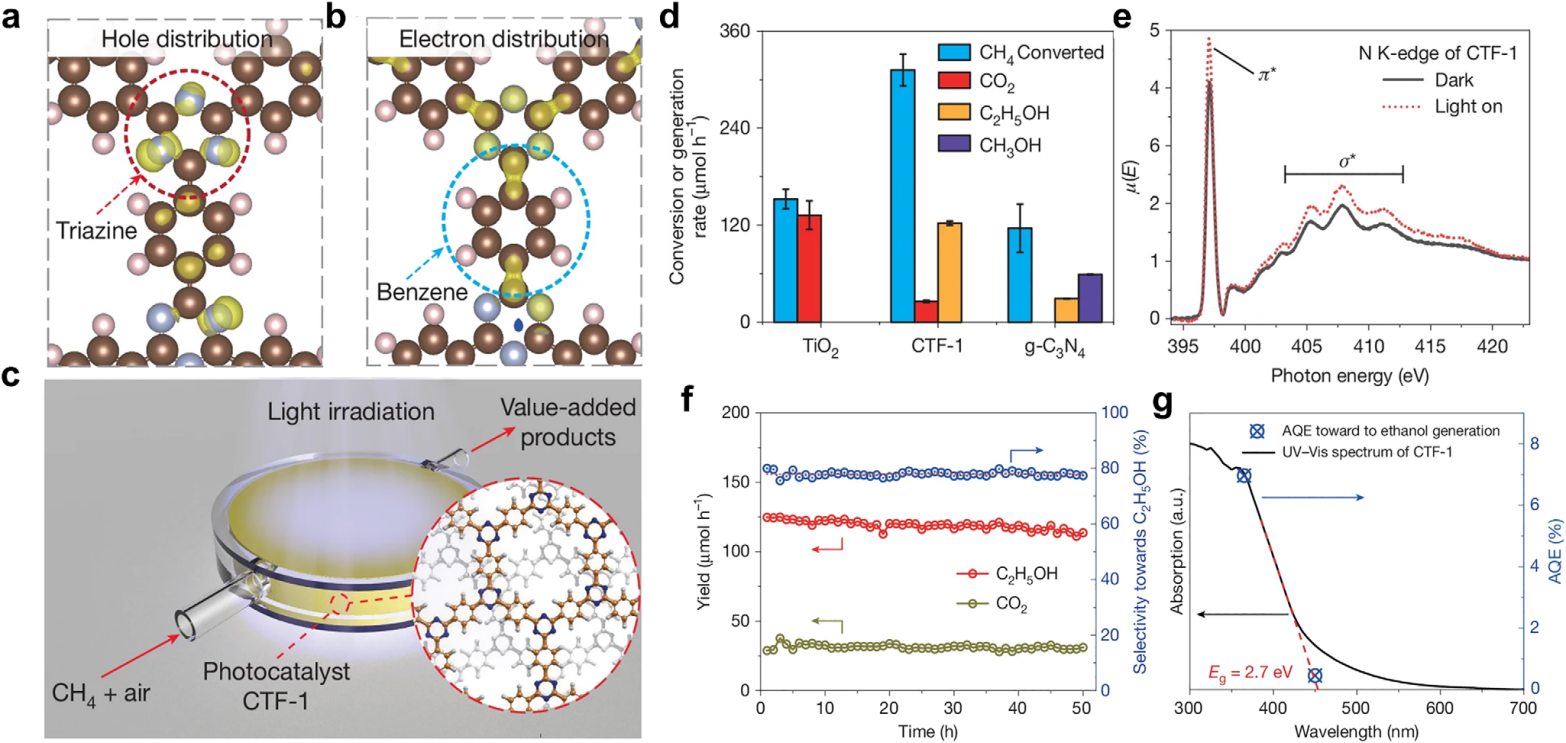
Spatial distribution of HOMO (a) and LUMO (b) of CTF‐1 catalyst. (c) Schematic representation of reaction system of photocatalytic methane oxidation to high‐value chemicals: reactor was sealed by a polytetrafluoroethylene screw‐type jacket, and the yellow part represents photocatalyst bed. (d) Comparison of photocatalytic activities of methane transformation on CTF‐1, g‐C_3_N_4_ and TiO_2_. (e) N *k*‐edge NEXAFS spectra of CTF‐1 in the presence or absence of LED light (320 nm) irradiation. (f) Long‐term product generation and C_2_H_5_OH selectivity during photocatalytic methane transformation by CTF‐1. Reaction conditions: 100 W 365 nm LED irradiation; dry gas flow rate  =  2,000 ml h^−1^, 16:1 methane (20% methane/argon) to oxygen (water‐saturated air, 20% oxygen/nitrogen) flow ratio. (g) AQE (blue dots) of photocatalytic methane transformation to ethanol by CTF‐1 under 365‐nm LED and 450‐nm LED irradiation. Ultraviolet–visible (UV–Vis) absorption spectrum (black line) of CTF‐1 was superimposed for comparison. The bandgap (*E*_g_) of CTF‐1 is presented as the intersection of the tangent to the absorption spectrum (red dashed line) with the wavelength axis. Reproduced with permission [185]. Copyright 2025, Springer Nature.

Molecular‐scale partitioning can also be encoded directly within a single crystalline framework. In a covalent triazine‐based system composed of alternating triazine and benzene motifs, intrinsic molecular heterojunctions are formed within a single crystalline lattice (Figure 13) [185]. These motifs promote charge separation and establish preferential adsorption domains for O_2_ and H_2_O. CH_4_ activation and C─C coupling occur in electronically differentiated regions that are partially decoupled from primary oxidation sites. Isotopic labeling confirms controlled ethanol formation with limited deep oxidation. Here, electronic separation and adsorption partitioning operate cooperatively to reduce overlap between activation and preservation processes.

Triphasic interfaces and membrane‐assisted configurations operate according to similar principles. Gas diffusion layers localize CH_4_ at activation sites while facilitating rapid removal of oxygenates from oxidative domains. Even when radical chemistry persists locally, a shorter residence time reduces the probability of secondary oxidation. Selectivity is stabilized through temporal control rather than through suppression of oxidative strength. This regime marks a qualitative shift in architectural design. Reactive intermediates are not only defined chemically but also constrained physically. Oxygenate preservation is encoded into the spatial organization of the catalyst. As a result, selectivity becomes less sensitive to fluctuations in radical concentration or light intensity. Nevertheless, several challenges remain. Quantitative mapping of intermediate concentration gradients within illuminated porous frameworks is rarely available. Direct measurements of CH_3_OH diffusion coefficients in confined domains and their correlation with local carrier density remain limited. Furthermore, long‐term structural stability under continuous flow and variable photon flux requires further investigation. Spatial partitioning represents the most advanced form of architectural synchronization in current photocatalytic CH_4_ conversion. It integrates reactive species control, carrier routing, and intermediate residence within a single spatial design, allowing selectivity to arise from controlled exposure rather than from kinetic compensation alone.

### 5.4 Remaining Architectural Gaps

The progression from radical‐dominated systems to coordination‐confined architectures and spatially partitioned designs shows that CH_4_ selectivity cannot be achieved by strengthening oxidation alone. Each architectural regime addresses one aspect of the activation–preservation asymmetry, yet none resolves it completely. In radical‐dominated systems, selectivity relies on maintaining a narrow kinetic balance. Reactive species are generated and consumed within the same spatial domain, where CH_4_ activation competes directly with oxygenate oxidation. Even moderate increases in radical flux can accelerate deep oxidation. Structural discrimination is minimal, leaving product distribution highly sensitive to illumination intensity, oxidant concentration, and transport conditions.

To overcome these limitations, coordination‐confined architectures redefine the reactive manifold by stabilizing bound oxygen intermediates. Hydrogen abstraction or oxygen insertion occurs within geometrically constrained sites, reducing indiscriminate radical propagation and improving pathway discrimination. However, activation and preservation remain spatially proximate. CH_3_OH generated at coordination pockets can still encounter accumulated carriers or secondary oxidants when the residence time is prolonged. Reactive species identity is programmed, but intermediate lifetime and spatial separation remain only partially controlled. Spatially partitioned systems introduce explicit separation between activation and preservation domains. Oxidant generation, carrier routing, and product evacuation are organized within defined interfaces or molecular heterojunctions. Oxygenate exposure time is shortened, and selectivity becomes less dependent on suppressing radical formation. Nevertheless, quantitative understanding of concentration gradients and carrier distribution within these architectures remains limited. Direct correlation among carrier density, reactive oxygen flux, and oxygenate lifetime is rarely established under CH_4_ reaction conditions.

These observations indicate that durable CH_4_ selectivity requires synchronization of thermodynamic moderation, coordination‐defined oxygen activation, directed carrier routing, and residence‐time control. Optimization of any single variable in isolation yields only conditional improvements. Increasing oxidation potential without confining reactive species accelerates overoxidation. Defining metal–oxo intermediates without regulating carrier accumulation creates localized oxidative hotspots. Enhancing mass transport without narrowing the oxidation window increases conversion while sacrificing selectivity.

Accordingly, future progress depends on integrating these variables within a unified architectural framework. Quantitative operando measurements capable of mapping reactive oxygen flux, intermediate lifetime, and spatial carrier distribution are necessary to establish predictive relationships. Design strategies must move beyond empirical optimization toward structural encoding of activation–preservation decoupling. Selective CH_4_ photoconversion does not arise from maximal oxidative strength, but from controlled exposure of reactive intermediates. Architectural design must therefore define where activation occurs, how oxygen is delivered, how carriers migrate, and how rapidly oxygenates depart. Only when these elements are coordinated can activation and preservation coexist without direct competition.

The architectural regimes discussed above also imply different selection criteria when the target product or reaction pathway changes. For selective partial oxidation to liquid oxygenates, the priority is activation–preservation decoupling. Suitable architectures should provide sufficient oxidative strength for C−H activation while restricting radical diffusion and shortening the residence time of oxygenated products. Coordination‐confined systems containing isolated metal sites, porphyrinic centers, or cocatalyst‐modified domains are thus preferred when surface‐bound M−OOH, M−O, or high valent M═O intermediates can be stabilized without excessive freely diffusing ^•^OH generation. In addition, microenvironments that promote CH_4_ accessibility and oxygenate desorption are desirable because they reduce the probability of secondary oxidation within highly oxidative domains.

In contrast, for oxidative coupling of CH_4_, the selection criteria shift from radical suppression to radical regulation. C─C bond formation requires the generation and coupling of methyl species, so radical or surface‐bound methyl chemistry should be controlled rather than eliminated. Architectures that confine radical formation within defined interfaces, moderate carrier flux, and maintain sufficient local methyl‐species concentration can promote coupling before deep oxidation occurs. In this case, radical lifetime, methyl‐species density, and diffusion geometry become more relevant descriptors than maximal oxidative strength.

For non‐oxidative CH_4_ coupling, the absence of oxygen‐derived oxidants shifts the design requirements toward direct C─H activation and charge utilization. Suitable organic photocatalysts should favor efficient exciton dissociation, localized charge accumulation, and high‐energy active sites capable of weakening or cleaving the C─H bond without relying on oxygen‐derived reactive species. Built‐in electric fields, strongly polarized donor–acceptor frameworks, metalated coordination sites, or plasmon‐coupled architectures may be beneficial, provided that catalyst degradation and charge recombination are suppressed. Accordingly, catalyst selection should be guided by the target reaction pathway rather than material category alone. Partial oxidation requires confined oxidation and product preservation; oxidative coupling requires controlled methyl‐species formation and C─C coupling; non‐oxidative coupling requires direct activation and efficient charge utilization. This pathway‐specific perspective extends the architectural framework from mechanistic interpretation toward practical catalyst selection.

**FIGURE 14:**
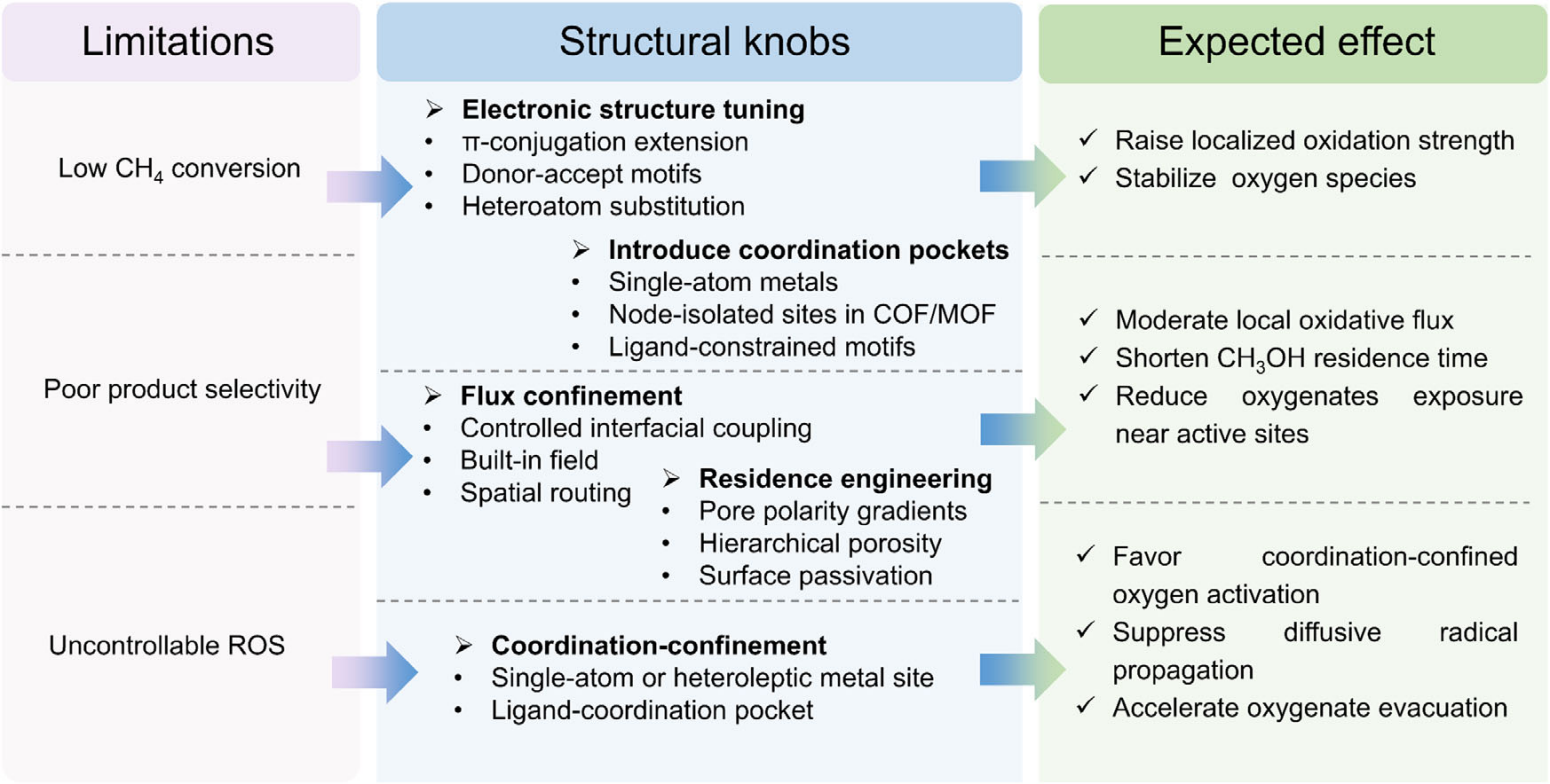
Structural design roadmap for selective CH_4_ photoconversion over organic semiconductor architectures.

Finally, these pathway‐dependent requirements can be further organized according to distinct kinetic regimes governing selective CH_4_ photooxidation, including activation‐limited, overoxidation‐limited, and radical‐dominated scenarios (Figure 14). Each regime is associated with a characteristic set of structural encoding strategies and kinetic consequences.

## 6 Design Principles and Outlook

Selective photocatalytic CH_4_ conversion is constrained by the intrinsic asymmetry between C─H activation and oxygenate preservation. Durable selectivity does not arise from stronger oxidation or higher carrier density alone, but from structural regulation of how reactive intermediates are generated, transported, and confined. Organic semiconductor architecture provides flexibility to encode this regulation within programmable frameworks. The remaining challenge is to convert architectural intuition into predictive design rules.

### 6.1 Reactive Oxygen Identity

Reactive species coexist dynamically under illumination. ^•^OH, O_2_^•−^, and peroxide intermediates can interconvert within the same catalytic environment, obscuring mechanistic interpretation. Pathway selectivity depends on the quantitative definition of oxidative species flux. Future progress requires measurement of ROS generation rates, steady‐state concentrations, diffusion lengths, and lifetimes under realistic CH_4_ conversion conditions. Operando electron paramagnetic resonance, time‐resolved optical spectroscopy, and isotope‐resolved oxygen tracing should be combined to correlate electronic polarization and coordination geometry with ROS distribution. Establishing direct relationships between oxidative species flux and product selectivity is essential for predictive control of CH_4_ oxidation pathways.

### 6.2 Activation–Preservation Balance

CH_4_ conversion operates within a narrow kinetic regime in which C─H activation and controlled oxygenate formation must be sustained simultaneously. Increasing oxidative driving force often accelerates deep oxidation, whereas excessive suppression of radical chemistry limits CH_4_ conversion. Structural solutions must dynamically regulate oxidation strength rather than simply increase or suppress it. Adaptive electronic states, modulation of PCET, and bias‐assisted photochemical operation offer potential routes to maintaining this balance. Multielectron oxygen‐activation pathways that minimize free radical release are particularly promising, as they reduce competition between CH_4_ activation and CH_3_OH degradation.

### 6.3 Microenvironment Engineering

Secondary oxidation frequently arises from the re‐encounter between oxygenates and ROS. Although pore confinement and reticular interface design can reduce exposure, a quantitative understanding of intermediate residence time remains limited. Coupling molecular simulation with transient spectroscopic probes can clarify how pore polarity, hydrogen‐bond networks, and local dielectric environments influence stabilization of methyl and CH_3_OH intermediates. Frameworks that preferentially adsorb CH_4_ while discouraging oxygenate re‐adsorption represent an important direction. Establishing correlations between adsorption thermodynamics, desorption kinetics, and oxygenate survival probability under steady‐state illumination will enable rational microenvironment design.

### 6.4 Charge Architecture

Selective multielectron oxygen activation requires sustained charge separation and directed carrier migration. High‐selectivity systems often operate under constrained irradiation conditions, indicating incomplete utilization of photogenerated carriers. Architectures that integrate internal electric fields, molecular heterojunctions, or well‐defined Z‐ and S‐type charge‐transfer motifs within organic frameworks provide a route to preserving redox strength while extending carrier lifetime. Translation into continuous‐flow and gas‐solid photoreactors will require coordination between catalyst architecture and reactor design to prevent oxidant dilution and product reoxidation.

### 6.5 Predictive Design

Recurring structural motifs in CN, MOF, and COF platforms indicate that CH_4_ photoconversion can be rationally programmed when electronic polarization, site isolation, and microenvironment confinement are treated as interdependent variables. Integration of first‐principles electronic structure calculations with systematic quantification of reactive oxygen flux and intermediate transport can enable predictive selection of framework topologies tailored to pathway‐specific CH_4_ conversion. Machine‐learning approaches that incorporate kinetic and thermodynamic descriptors may further accelerate discovery. Selective CH_4_ photoconversion in organic semiconductor systems is thus governed by structural control over charge accumulation, oxygen activation geometry, and intermediate migration. Encoding multiscale regulation within hierarchical architecture provides a viable route toward stable, scalable CH_4_ valorization.

## Conflicts of Interest

The authors declare no conflicts of interest.

## Data Availability

Data sharing not applicable to this article as no datasets were generated or analysed during the current study.
